# Molecular Evolution and Diversity of *Conus* Peptide Toxins, as Revealed by Gene Structure and Intron Sequence Analyses

**DOI:** 10.1371/journal.pone.0082495

**Published:** 2013-12-13

**Authors:** Yun Wu, Lei Wang, Maojun Zhou, Yuwen You, Xiaoyan Zhu, Yuanyuan Qiang, Mengying Qin, Shaonan Luo, Zhenghua Ren, Anlong Xu

**Affiliations:** 1 State Key Laboratory of Biocontrol, Guangdong Province Key Laboratory of Pharmaceutical Functional Genes, National Engineering Research Center of South China Sea Marine Biotechnology, Department of Biochemistry, College of Life Sciences, Sun Yat-sen University, Guangzhou, China; 2 Beijing University of Chinese Medicine, Beijing, China; University of California, Los Angeles, United States of America

## Abstract

Cone snails, which are predatory marine gastropods, produce a cocktail of venoms used for predation, defense and competition. The major venom component, conotoxin, has received significant attention because it is useful in neuroscience research, drug development and molecular diversity studies. In this study, we report the genomic characterization of nine conotoxin gene superfamilies from 18 *Conus* species and investigate the relationships among conotoxin gene structure, molecular evolution and diversity. The I1, I2, M, O2, O3, P, S, and T superfamily precursors all contain three exons and two introns, while A superfamily members contain two exons and one intron. The introns are conserved within a certain gene superfamily, and also conserved across different *Conus* species, but divergent among different superfamilies. The intronic sequences contain many simple repeat sequences and regulatory elements that may influence conotoxin gene expression. Furthermore, due to the unique gene structure of conotoxins, the base substitution rates and the number of positively selected sites vary greatly among exons. Many more point mutations and trinucleotide indels were observed in the mature peptide exon than in the other exons. In addition, the first example of alternative splicing in conotoxin genes was found. These results suggest that the diversity of conotoxin genes has been shaped by point mutations and indels, as well as rare gene recombination or alternative splicing events, and that the unique gene structures could have made a contribution to the evolution of conotoxin genes.

## Introduction

The genus *Conus*, which contains >500 species, is one of the largest genera of marine invertebrates [1]. Cone snails can produce toxic venoms for prey capture, defense, or competitor deterrence. Most cone snails are specialist predators, feeding predominantly on worms (vermivorous), fish (piscivorous), or other marine gastropods (molluscivorous) [2]. Based on mitochondrial 16S rRNA sequence data, 17 phylogenetic clades of *Conus* species have been defined. Clades I–IV comprise piscivorous species, V and VI comprise molluscivorous species, and the remaining clades are vermivorous [3].

The dominant components of cone snail venoms are small peptide toxins typically comprising 12∼50 residues and 1–5 disulfide bridges, which have become known as conotoxins or conopeptides [1]. There are approximately 100∼1000 different conotoxins per *Conus* species, and it has been estimated that >100,000 different pharmacologically active components are present in the venoms of all living cone snails [4], [5]. The conotoxins can be broadly divided into the disulfide-rich conopeptides, which contain two or more disulfides, and disulfide-poor conopeptides, with zero or one disulfide bond. Other classification schemes have also been used to describe different aspects of conotoxins, such as the “gene superfamily”, the “cysteine framework” and the “pharmacological family” classification schemes [6]. Among these, the “gene superfamily” classification scheme maybe the most popular, and it can be applied to nearly all conotoxins [6].

Each gene superfamily is defined by a highly conserved signal sequence in the precursor, and this classification scheme focuses on the evolutionary relationships among conopeptides [6]. At present, 26 conotoxin superfamilies have been defined, and new ones are regularly being discovered [7]. The signal peptides of each superfamily have little homology with one another. The A, M, O1 and T superfamilies are the four biggest groups, and each included more than 100 toxins [6], [7]. Most of the well-studied conotoxins with effective bioactivities are members of these superfamilies. For example, omega-conotoxin MVIIA, which belongs to the O1 superfamily, has been approved by the FDA for use in severe pain [8]. Certain superfamilies have very few members, such as the L, V and Y superfamilies [9], [10], [11]. The bioactivities of the majority of those toxins remain unknown.

To date, there have been a number of studies reporting the cloning of cDNAs encoding conotoxins, but there are few reports available on the characterization of conotoxin genes and their gene structures. A study characterizing the *Conus bullatus* genome shows that ∼7% of the total length of the genome has been obtained, and the genome-wide base composition, simple repeat content, and mobile element densities were also analyzed [12]. However, no information regarding the conotoxin genes was reported in that study. The structural organizations of several O1 and A conotoxin precursors have been found to be different from each other. The former consist of three exons and two introns, and the latter consist of two exons and one intron [13], [14], [15]. The gene structures of the remaining 24 superfamilies (other than the O1 and A superfamilies) have not yet been studied.

In this report, we present the gene structures of nine conotoxin superfamilies from 18 different *Conus* species with different feeding habits. Eight of the superfamilies have similar architectures, with two introns and three exons, while the A superfamily contains one intron and two exons. The diversity of gene structures of conotoxin superfamilies may be related to the evolution of *Conus* species. The introns are conserved in a single superfamily but divergent among different superfamilies. We uncovered the first evidence of splicing variation in conotoxins. Our analysis of the diversity of conotoxins suggests that their unique gene structure has likely played a certain role in the evolution of conotoxin genes.

## Results

### The gene structures of conotoxin precursors

First, the coding sequences of I1, I2, M, O2, O3, P, S, and T superfamily conotoxins were isolated from cDNA libraries of 18 *Conus* species. Highly abundant sequences from each superfamily were selected as the templates for genome walking (GenBank accession numbers JX293408 - JX293558) (Table 1). The gene structures of all eight superfamilies have similar architecture, containing two introns and three exons, similar to those of the O1 conotoxin genes [13].

**Table 1:** Conotoxins selected for genome walking.

Superfamily	Framework	Toxin	*Conus* species	Toxin precursor	GenBank number
I1	XI	Tx11.3	*C. textile*	MKLCVTFLLVLVILPSVT *GVKSSERTLSGAALRGDR* **GTCSGRGQECKHDSDCCGHLCCAGITCQFTYIPCK.**	JX293528
I2	XII	TxX	*C. textile*	MSGHTSVSFLLLSIVALGMVAT **VICSCDSEFSSEFCERPEESCSCSTHTCCHWARRDQCMKPQ RCISAQKGN** *GRRRLIHMQK.*	JX293530
M	III	Tx3-KP01	*C. textile*	MMSKLGVLLTICLLLFPHTAVPLDG *DQPADQPAERLQDDISSEHHPMFNSIRR* REQNQFKSFTSVKLLDSRGER **CCGPTACLAGCKPCCG.**	JX293525
O2	VI/VII	Tx7.29	*C. textile*	MEKLTILLLVAAVLMSTQA*LIQ DQRQKAKINLFSKRQAYAR* **DW WDDGCSVWGPCTVNAECCSGDCHETC.**	JX293557
	XV	Tx15a	*C. textile*	MEKLTILILVATVLLAIQVLVQS*DG EKPLKGRVKQNAAKRLWVHMKG PR* **LCTPRNEPCYEDGECCPNLECRCRTVADCQAGYKCRV.**	JX293529
O3	VI/VII	S6.16	*C. striatus*	MSGLGIMVLTLLLLVFMATS*RQDAG EKQATQRDAISVIGRRSIIRRR*V **DEECNEICGEQGKNCCGRSNGTPRCAKVCFG.**	JX293408
P	IX	Tx9a	*C. textile*	MHLSLARSAVLMLLLLFALGNFV VVQS*GQITRDVDNGQLTDNRRNLQSKW KPVSLYMSRR* **GCNNSCQEHSDCESHCICTFRGCGAVNG.**	JX293527
S	VIII	Tx8.1	*C. textile*	MMLKMGAMFVLLLLFTLASS*QQEG DIQARKTHLKSGFYRTLPR FAR* **GCTISCGYEDNRCQGECHCPGKTNCYCTSGHHNKGCGCAC.**	JX293526
T	V	TeAr193	*C. textile*	MRCLPVFVILLLLIASAPSVDA *QPKTKDDIPQASFL DNAKRYLQVLESKR* **NCCRRQICCGRTK.**	JX293555
		Tr5.1	*C. terebra*	MRCLPVFIILLLLILPAPSADV *QPKTKDHVHLASFL DSAKRTVR* **GHCCPYYPQCCPSG.**	JX293524
		Tx5.11	*C. textile*	MLCLPVFIIVLLLIASAPSFDA *RSKTKDDVPLSSFR DNAKRILQTLQTKR* **DCCKGNPGCCGWD.**	JX293556
	X	S10.1	*C. striatus*	MRCLPVFVILLLLIASAPSVDA *QLKTKDDVPLSSFR GHAKSTLRRLQDK* **QTCCGYRMCIPCG.**	JX293409

The conotoxin precursors are separated according to different exons. Signal peptides are shown in uppercase letters, propeptides are in italics, mature peptides are in boldface, and the extra propeptide of Tx3-KP01 is underlined.

**Figure 1:**
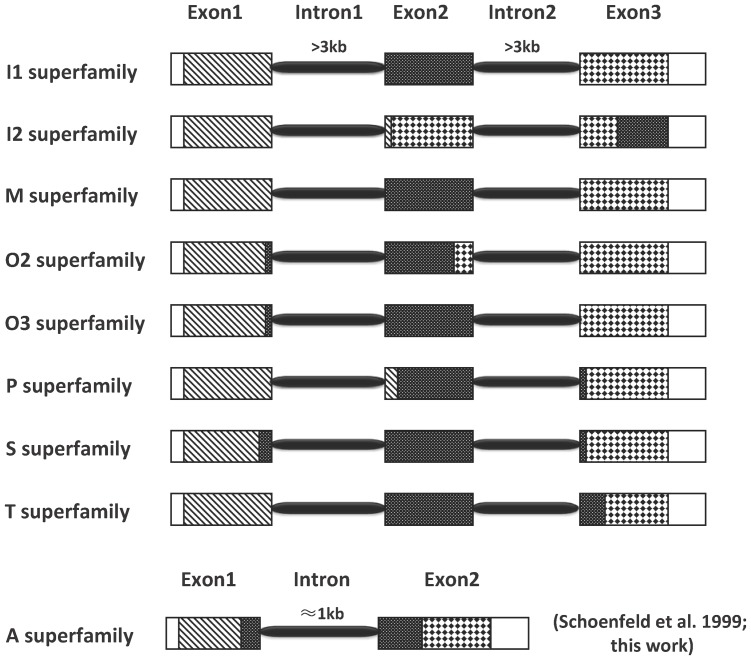
Gene structures of nine conotoxin superfamilies. Different segments of the toxin sequence are marked with different boxes. 5′ or 3′UTR: white box; signal sequence: hatched box; propeptide and postpeptide: stippled box; mature sequence: diamond box.

The corresponding cDNA sequences were analyzed to determine the exon/intron junctions. The identified junctions are typical donor and acceptor splice sites that have followed the GT/AG rule. All introns are longer than 3 kb, similar to the sizes of the introns of O1 superfamily genes [13]. The three exons roughly correspond to the signal sequence, pro-region and mature sequence, with some differences in the number of nucleotides at the boundaries. Differences were also observed among the superfamilies (Figure 1). Intron1 of all but the I2 superfamily, which has a phase 0-intron, are phase 1 introns; and intron2 of all but the I2 and T superfamilies, which have phase 1-introns, are phase 2 introns [16]. The sequences of the intron1 and intron2 from the same superfamily are poorly conserved (Figure S1).

### A superfamily

In the A superfamily, there is only one intron inserted in the pro-region, dividing the conotoxin precursor into two exons [13], [15]. The intron is approximately 1 kb in length, allowing the sequence of the precursor to be cloned by simple gPCR.

A total of 24 A superfamily conotoxin gene sequences were obtained from the genomes of *C. varius, C. terebra, C. emaciatus, C. caracteristicus, C. betulinus* and *C. striatus* (Table S1). Among them, the intronic sequence of α4/4 and framework II conotoxins were not known prior to this study. A comparison of the 24 new sequences and four known A superfamily introns [GenBank: Ac1.1b, DQ311055; Mr1.2, DQ309774; Lp1.4, DQ311056; Pu1.1, DQ309775.] [15] showed that, although these introns vary in length (0.9-1.4 kb), the beginning and ending portions of the intronic sequences were conserved (Figure S2). The high conservation of the beginning and ending of these introns may play a role in the intron splicing mechanism during transcription. Certain toxins contained the same encoding region, such as Tr1.1, Bt1.7, S1.1 and S1.10, but their introns exhibited some differences in length (Table S1). It has been suggested that some conotoxin genes are present in multiple copies in the genome.

After aligning the intron sequences, we created a percent identity matrix of the sequences in ClustalX 2.0 (Table S2). The introns from the same *Conus* species contain highly homologous sequences; for example, Ec1.7 and Ec1.8b share up to 98% sequence identity, and Ca1.6a and Ca1.7c share up to 99% identity. The four toxins from *C. striatus* (S1.1, S1.10, SII and SIVA), have different cysteine scaffolds in their mature peptides, but their intron sequences share greater than 84% identity. The introns from species within the same phylogenetic clades had higher identities to one another than to introns from species in different clades [3]. *C. striatus* and *C. achatinus*, *C. terebra* and *C. emaciatus*, *C. caracteristicus* and *C. pulicarius* belong to Clade I, Clade XIII and Clade XIV, respectively. The identities between these intron pairs are all >82%. Bt1.7b of Clade X shared a relatively low sequence identity with the other introns, so did Mr1.2, the only intron obtained from molluscivorous species. Therefore, the intron sequences could be used as genetic markers for molecular evolution studies in some cases.

### I1 superfamily

Three new I1 conotoxin genes were cloned from the cDNA libraries of *C. textile* and *C. striatus*. Tx11.3 was used as the template for genome walking to identify the gene structures. The three exons of this superfamily correspond almost perfectly to the signal sequence, pro-region and mature sequence. Exon1 encodes the end of the 5′UTR and all but the last 2 bp of the signal sequence; exon2 encodes the last 2 bp of the signal sequence and all but the last 1 bp of the pro-region; and exon3 encodes only 1 bp of the pro-region, the mature sequence and the 3′UTR.

### I2 superfamily

Unlike those of the other superfamilies, the precursors of I2-conotoxins have a signal peptide that is followed immediately by the mature peptide. Instead of an intervening propeptide, the precursors include a short C-terminal extension, which is called the postpeptide [17]. A framework XII conotoxin TxX obtained from *C. textile* was selected as the subject for the genome walking [18]. Exon1 of TxX encodes the end of the 5′UTR and the first 66 bp of the signal sequence; exon2 encodes the last 9 bp of the signal sequence and all but the last 29 bp of the mature sequence; and exon3 encodes the remaining mature sequence, the postpeptide sequence and the 3′UTR.

### O2 superfamily

The O2 superfamily proteins have two cysteine scaffolds: framework VI/VII and XV [19], [20]. Here, 22 new O2 superfamily conotoxin genes were isolated; six of these belong to framework VI/VII, and the others belong to framework XV. Two toxins, Tx7.29 and Tx15a, which were both obtained from the cDNA library of *C. textile* but had different frameworks, were selected as subjects for genome walking. Two long introns divided the coding region into three exons for each gene, but the intron splice sites differ between the two toxins. For Tx7.29, exon1 contains the last portion of the 5′UTR, the signal sequence and the first 10 bp of the pro-region; exon2 contains all but the first 10 bp of pro-region and the first 5 bp of the mature sequence; and exon3 contains all but the first 5 bp of mature sequence and the 3′UTR. For Tx15a, exon1 contains the last part of the 5′UTR, the signal sequence and the first 7 bp of the pro-region; exon2 contains all but the first and last 7 bp of the pro-region; and exon3 contains the last 7 bp of the pro-region, the mature sequence and the 3′UTR.

### O3 superfamily

The O3 superfamily conotoxins also shared cysteine framework VI/VII [19]. Seven new O3-toxin sequences were obtained. S6.16 was selected as the template for genome walking. Exon1 encodes the end of the 5′UTR, the signal sequence and the first 16 bp of the pro-region; exon2 encodes the remaining portion of the pro-region and the first 2 bp of the mature sequence; and exon3 encodes the remaining mature sequence and the 3′UTR.

### P superfamily

To date, only nine P superfamily conotoxins have been identified, and they all belong to framework IX (C-C-C-C-C-C) [21]. Tx9a was selected as the template for genome walking. In contrast to those of the other superfamilies, exon1 of Tx9a contains the end of the 5′UTR and only part of the signal sequence; exon2 contains the last 11 bp of the signal sequence and part of the pro-region; and exon3 contains the remaining 31 bp of the pro-region, the mature sequence and the 3′UTR.

### S superfamily

The S superfamily only includes 17 toxins, all of which have been classified as framework VIII (C-C-C-C-C-C-C-C-C-C) [22]. Two new toxin genes were cloned from the cDNA library of *C. striatus*. The complete precursor sequence of Tx8.1 was also obtained and was selected as the template for the gene structure study. Exon1 of Tx8.1 encodes the last part of the 5′UTR, the signal sequence and the first 13 bp of the pro-region; exon2 encodes the all but first 13 bp and last 10 bp of the pro-region; and exon3 encodes the last 10 bp of the pro-region, the mature sequence and the 3′UTR.

### T superfamily

The two main cysteine scaffold types in the T superfamily are framework V and X [2]. In this study, 16 new T-toxin genes were isolated; 15 of these belong to framework V (CC-CC), and only one toxin, S10.1, belongs to framework X (CC-CXPC). Three toxins (TeAr193, Tr5.1 and S10.1) from different species were selected for genome walking, and all of these have the same gene structures. Exon1 contains the last portion of the 5′UTR and all but the last 2 bp of the signal sequence; exon2 contains the last 2 bp of the signal sequence and 43 bp of the pro-region; and exon3 contains the remaining part of the pro-region, the mature sequence and the 3′UTR.

In previous reports, T superfamily was separated into two groups, the MRCL and MLCL groups, according to their signal peptides [19]. We cloned a new conotoxin, Tx5.11, from the MLCL group for genome walking. The gene structure and position of the introns in Tx5.11 were the same as those of other T conotoxins. It seems that both MRCL- and MLCL-group T superfamily conotoxins originated from the same ancestor. The first and last segments of the two introns are conserved within the T superfamily and across different *Conus* species, consistent with the results observed in the O1, A, and M superfamilies.

### GC content

We determined the GC content of all intron and exon sequences from the amplified conotoxins. The estimated GC contents are 41.83% for the introns, 50.65% for the exons, and 42.76% for the entire gene. The GC content of the introns is lower than that of the exons. In *Conus textile* alone, the GC contents of the introns and exons sequences were estimated as 43.33% and 48.64%, respectively. The overall GC content of the *Conus textile* genome is 43.75%, which is similar to that previously reported for the *Conus bullatus* genome (42.88%) [12].

### Alternative splicing

**Figure 2:**
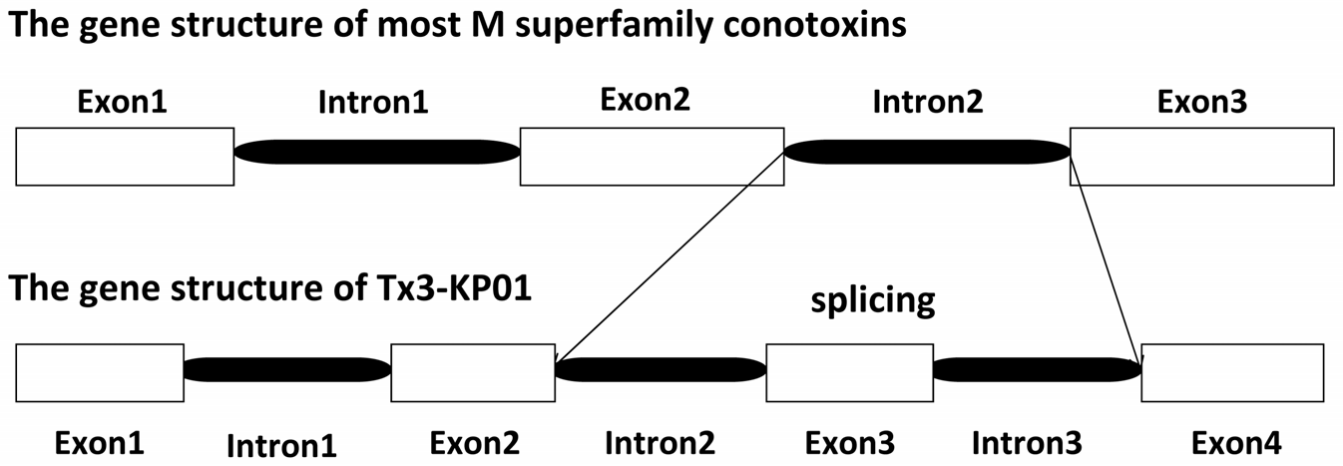
Alternative splicing of the M superfamily conotoxin Tx3-KP01. Intron2 was divided into two new introns and a new exon.

Eight toxins from the two groups of the M superfamily (MMSK- and MLKM-group) had previously been selected as subjects for genome walking (unpublished data). One of these genes, Tx3-KP01, which belongs to the MMSK group, showed an additional 63 bp of sequence in its pro-region. We found that this region was a new exon produced by the alternative splicing of intron2, which was divided into two new introns and a new exon (Figure 2). This is the first demonstration of splice site variation in conotoxin genes.

### Intron and gene expression

**Figure 3:**
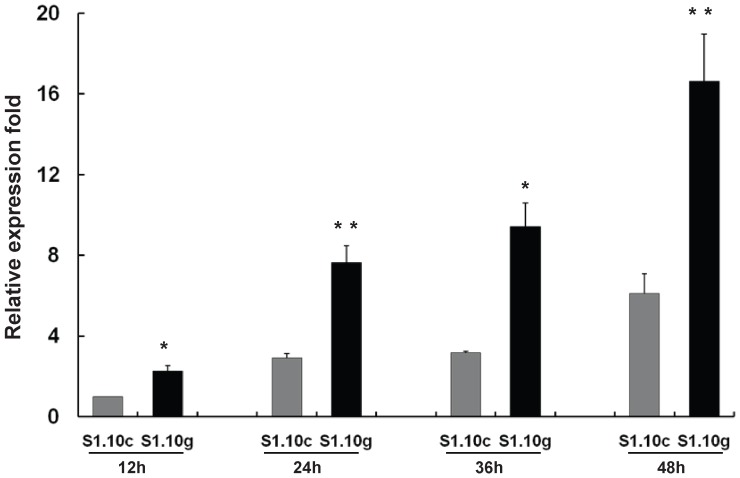
Expression profiles of S1.10-cDNA and S1.10-gDNA. The mRNAs were sampled at 12, 24, 36 and 48± SD (n = 3). *, p<0.05; **, p<0.01.

To explore the potential effects of intron sequences on the expression of conotoxin genes, we constructed two eukaryotic expression vectors encoding A-conotoxin S1.10: one intron-free construct (S1.10-cDNA) and one intron-containing construct (S1.10-gDNA). Real-time PCR showed that the relative expression of S1.10-gDNA as significantly higher than that of S1.10-cDNA at 12, 24, 36 and 48 hours after transfection (Figure 3). The pFXB vector is over-expressed in cells, and the promoter elements of the two genes are identical. Therefore, the higher expression of S1.10-gDNA implied that the presence of the intron enhances the transcriptional efficiency or mRNA stability of conotoxin genes, as has been reported previously for other genes [23].

## Discussion

### Conotoxin gene structures are correlated to the gene superfamily only

As mentioned above, three classification schemes have been used to define conotoxins: the “gene superfamily”, the “cysteine framework” and the “pharmacological family”. The relationships between conotoxin gene structures and classification schemes are shown in Table 2. All but the A superfamily genes share the same gene structure, with three exons and two introns.

**Table 2:** The relationships among conotoxin gene structure, gene superfamily, cysteine framework and pharmacological family. The correlations among the classification schemes have also been showed by Kaas et al [7].

Gene superfamily	Cysteine framework	Pharmacological family	Gene structure of precursor	Reference
A	I, II, IV	α, κ, ρ	2 exons + 1 intron	Schoenfeld et al. 1999; This work
I1	XI	ι	3 exons + 2 introns	This work
I2	XII		3 exons + 2 introns	This work
M	III, XVI	α, κ, μ, ι	3 exons + 2 introns	This work
O1	VI/VII	γ, δ, μ, ω, κ	3 exons + 2 introns	Schoenfeld et al. 1999
O2	VI/VII, XV	γ	3 exons + 2 introns	This work
O3	VI/VII		3 exons + 2 introns	This work
P	IX		3 exons + 2 introns	This work
S	VIII	α, σ	3 exons + 2 introns	This work
T	V, X	χ, ε, μ	3 exons + 2 introns	This work

The O1, O2 and O3 superfamilies all contain framework VI/VII conotoxins, but the framework VI/VII toxins in the three superfamilies have different intronic sequences. In contrast, the conotoxins with different frameworks as framework V and X conotoxins belonging to the T superfamily exhibits conserved intronic sequences. The α-pharmacological family conotoxins are selective antagonists of the nicotinic acetylcholine receptors [24]. α-conotoxins can be found in the A, M and S superfamilies; however, these superfamilies have different gene structures and introns. The A superfamily includes three cysteine frameworks (I, II and IV), and framework I can be further divided into several branches, i.e., the α3/5, α4/4 and α4/7 subfamilies. Most framework I and II toxins target nicotinic acetylcholine receptors and thus belong to the α-pharmacological family, but several framework IV toxins that can cause nerves to fire uncontrollably [25] belong to the κ-pharmacological family. Although these genes have the same gene structure and conserved intron sequences, they have different functions.

Therefore, our data have confirmed that conotoxin gene structure is related to the gene superfamily only and does not closely follow the cyeteine framework or the pharmacological family. In contrast, conotoxin function is generally related to its cysteine scaffold. Therefore, it is possible to clone new conotoxin sequences of a certain superfamily by gPCR using primers corresponding to the 3′ end of the intron preceding the mature peptide region and the 3′UTR. However, the biological activity of a new toxin cannot be determined based on its gene sequence alone.

### The diversity of conotoxin superfamily gene structures may be related to the evolution of *Conus* species

Why do most conotoxin superfamily genes have two introns, while A superfamily genes have only one intron? The A superfamily is a relatively recent innovation compared to the superfamilies with two introns (Table 3) [26]. Cone snails, terebrids and turrids are traditionally assigned to the superfamily Conoidea. All of these species feed mainly on worms, whereas some species of cone snails can prey on fish and mollusks [27]. Some O-like, I2-like and P-type toxins have been isolated from terebrids and turrids [27], [28], [29], [30]. And I1-, O-, P-, S- and T-conotoxins have been cloned from *Conus californicus*, which is distant from the majority of other cone snails [31]. While A-conotoxins have not been found in *Conus californicus*, terebrids or turrids [27], [30], [31], [32]. In fact, although A superfamily toxins are present in the majority of *Conus* species, the A-α3/5 and A-framework IV subfamilies of conotoxins only co-exist in the piscivorous cone snails, which are believed to have arisen from the vermivorous *Conus* [33]. Those superfamilies with two introns are distributed more broadly across the Conoidea than the A superfamily and therefore should be derived from the more ancient species. While the A superfamily conotoxins should be *Conus* specific, their generation is closely related to the *Conus* species evolution. Thus, the gene structure of A superfamily conotoxins is unique. In addition, a previous report mentioned that some of the smaller conotoxin superfamilies have no introns at all [15]. These superfamilies were most likely generated even later than the A superfamily. Due to the lack of template sequences, the gene structures of some small superfamilies could not be determined. However, some of them (such as the L, V and Y superfamilies) have been obtained from only one or two vermivorous cone snails [6]. These genes may be associated with the particular prey of those *Conus* species.

**Table 3:** Conotoxin superfamily distributions within selected Conoidea.

				Superfamily															
				One intron				Two introns							unknown				
				A-α3/5	A-α4/4	A-α4/7	A-IV	I1	I2	M	O	P	S	T	D	J	L	V	Y
Conoidea	Cone snails	Major species	Piscivorous	**✓**	**✓**	**✓**	**✓**	**✓**	**✓**	**✓**	**✓**		**✓**	**✓**		**✓**			
			Molluscivorous		**✓**	**✓**		**✓**	**✓**	**✓**	**✓**	**✓**	**✓**	**✓**					
			Vermivorous		**✓**	**✓**		**✓**	**✓**	**✓**	**✓**	**✓**	**✓**	**✓**	**✓**	**✓**	**✓**	**✓**	**✓**
		*Conus californicus*						**✓**			**✓**	**✓**	**✓**	**✓**		**✓**			
	Terebrids	*Hastula hectica*									**✓**	**✓**							
		*Terebra subulata*						**✓**			**✓**								
	Turrids	*Gemmula speciosa*										**✓**							
		*Lophiotoma olangoensis*							**✓**		**✓**	**✓**							
		*Polystira albida*										**✓**							

The checkmarks indicate the presence of the corresponding superfamily.

### Intron sequences may affect conotoxin gene expression

The comparison of the intronic sequences of eight superfamilies showed that although the intron sequences are conserved within a superfamily or at least a group, they are divergent among different superfamilies, except that all are flanked by a GT/AG donor–acceptor pair (Figure S1). One notable feature of these introns is their simple repetitive sequence contents. The introns contain stretches of the dinucleotide, trinucleotide or tetranucleotide repeats “GT”, “GA”, “CA”, “CT”, “CAT”, “CTGT”, “TACA”, and “TGCG”. Most eukaryotic genomes have a great number of different types of simple tandem repeats known as microsatellite DNA or simple sequence repeats [34]. The intronic microsatellites may affect gene transcription, mRNA splicing, or mRNA export to the cytoplasm [35]. For instance, a (TCAT)n repeat located in intron1 of the tyrosine hydroxylase (TH) gene acts as a transcription regulatory element *in vitro* [36], and a GT repeat in intron 2 of the human Na^+^/Ca^2+^ exchanger 1 gene (NCX1) is required for splicing activation and the regulation of NCX1 expression [37]. For conotoxins, these intronic simple repeats may be related to the transcriptional efficiency or mRNA stability of toxin genes.

A number of elements that regulate gene expression have been found in intronic sequences [38]. Introns have been shown to enhance or repress the transcriptional efficiency of many genes in varied organisms. Furthermore, intron splicing interacts with many pre-mRNA processing events, including 5′-end capping, 3′-end cleavage and polyadenylation, and sometimes RNA editing. There are hundreds of different conotoxins in the *Conus* venom, and the expression levels of different conotoxin transcripts can differ by orders of magnitude [39]. Our Real-time PCR results showed that the intron sequences of conotoxins may regulate the expression of these toxins (Fig. 3). Thus we analyzed the regulatory sequences in all the conotoxin introns using the TRANSFAC database [40]. Binding sites for SP1/3 [41], CATA-1 [42] and NF-1 [43] were found in different introns. These regulatory elements are mostly correlated with gene transcription. The actual functions of these theoretical transcription factor binding sites need to be verified in future studies.

### Gene recombination and alternative splicing may underlie the high diversity of conotoxin genes

The presence of introns in a conotoxin gene provides the opportunity for gene recombination, alternative splicing or trans-splicing events [44]. All these events may increase the diversity of conotoxin genes. Espiritu et al. reported that a novel δ-conotoxin may have arisen through the recombination of two parental δ-conotoxin genes [3]. Other studies have indicated that trans-splicing does not occur in mollusks [45], [46]. Alternative splicing of conotoxin genes was described for the first time in the present study.

Ordinarily, the cysteine framework and the gene superfamily of conotoxins essentially correspond to each other. One conotoxin from a certain superfamily may often belong to one or two cysteine frameworks. However, some conotoxin with new frameworks have arose in a given gene superfamily (Table 4). For example, the M superfamily conotoxins, most of which are classified as framework III, also include framework II, IV, VI/VII, IX and XIV toxins. The existence of these specific conotoxins may be due to point mutations and indels or gene recombination. Point mutations and indels in conotoxins are common [14], [19], [47]. However, if these events lead to the production of new toxins, all the intermediates must be selected through evolution. Recently, several conotoxins with odd number of cysteines which reported by Dutertre et al, could be intermediates for those specific conotoxins [36]. But these unique toxins also can be produced by the recombination of conotoxin genes. In general, the segments involved in recombination events are fairly large (700 to 2500 bp), and the length of conotoxin introns satisfies the requirements for recombination. Recombination would allow the mature peptide exon to be connected to a different signal peptide exon or propeptide exon, thus altering how the toxin is treated posttranslationally and possibly affecting its function.

**Table 4:** Conotoxins that may be involved in gene recombination events.

Conotoxin	Superfamily	Signal peptide	Framework	Mature peptide	GenBank number
SII	A	MGMRMMFTVFLLVVLATTVVS	II	GCCCNPACGPNYGCGTSCS	AAN77902
Cp2-DD02	M	MMSKLGVLVTICLLLFPLTAFG	II	NSASLISSWVDNTNFCCCSHDCATICDDCF	AEX60264
VxII	M	MMSKLGVLVTICLLLFPLTALPLDG	II	WIDPSHYCCCGGGCTDDCVNC	AAN78279
Ca4-VRS01	M	MGVVLFTFLVLFPLATLQLDA	IV	YECCVWPYCDGGCSSCVRSCE	AEX60241
Im6.1	M	MSKLGVVLFTLLLLVPLVTP	VI/VII	TCDPYYCNDGKVCCPEYPTCGDSTGKLICVRVTD	ACI96055
Im5.3	M	MLKVGVVLLIFLVLMSSA	IX	CIVGTPCHVCRSQSKSCNGWLGKQRYCGYC	ADZ74142
Bt14-H01	M	MLKMGVVLFTFLVLFPLATLQLDA	XIV	VWCDWEWCYGDCHCFD	AEX60163
Pu14.1	A	MGMRMMFAVFLLVVLATTVVS	XIV	VLEKDCPPHPVPGMHKCVCLKTC	ACL13206
Pu14.2	A	MGMRMMFTVFLLVVLATTVDS	XIV	DAEVVSTESDVIVTCEPCMNPACGPNYGKC	ACL13207
Pu14.3	A	MGMRMMFTVFLLVVLATTVDS	XIV	DSAAMHTEYDVIATDNCIPCSHPACGINRGKC	ACL13208

Alternative splicing is consistently observed in the transcription of eukaryotic genes and has also been reported in snake and scorpion venom peptides [48], [49], [50]. Alternative splicing in conotoxins is generally accompanied by a change in the length of the propeptide sequence. With regard to the conotoxin Pu14.5 reported by Lluisma et al, the propeptide of one of its precursors is obviously longer than others [51]. Those conotoxins with especially long or short propeptides might be generated by alternative splicing events. Similar to recombination events, a change in the propeptide sequence could also influence conotoxin bioactivity.

### Different exons have significantly different base substitution rates

The signal peptides which are usually only need to have a general hydrophobic character to function as secretion signals, are unexpectedly highly conserved [52]. The role of the propeptide has not yet been determined, but it may be related to folding and posttranslational modification [53], [54], [55]. In contrast, the mature peptides are extremely diverse [52]. The conservation of the signal peptide and the diversity of the mature peptide are inconsistent, suggesting that these elements were distributed in different exons due to the presence of introns and thus were generated through different evolution mechanisms.

For six conotoxin superfamilies, we computed the overall mean distance for nonsynonymous substitutions per nonsynonymous site (*Dn*) and synonymous substitutions per synonymous site (*Ds*) of each exon sequence, excluding the UTRs (Table 5, Text S1). By aligning the cDNA sequences of each superfamily, we found that the lengths of exon1 and exon2 are relatively stable and that insertions and deletions (indels) were rarely found in these elements. However, indels are commonly found in exon3, especially trinucleotide indels, resulting in a wide variety of lengths in exon3. To obtain the optimal alignment of the exon3 sequences, we deleted all gaps and missing data. Therefore, the actual *Dn* and *Ds* values of exon3 are higher than the values shown in Table 5. All the conotoxin sequences for analysis are listed in the Supporting Information.

**Table 5:** *Dn* (top) and *Ds* (bottom) values of each exon sequence within six conotoxin superfamilies.

	Superfamily								
Exon	A-α3/5	A-α4/4	A-α4/7	A-IV	I1	O2-VI/VII	O3	S	T
Exon1	0.052	0.092	0.080	0.022	0.066	0.086	0.067	0.045	0.082
	0.040	0.059	0.108	0.009	0.113	0.095	0.057	0.000	0.129
Exon2	0.160	0.467	0.427	0.194	0.272	0.308	0.121	0.190	0.242
	0.066	0.270	0.309	0.087	0.065	0.260	0.074	0.126	0.116
Exon3	-	-	-	-	0.495	0.526	0.345	0.506	0.392
	-	-	-	-	0.382	0.680	0.340	0.402	0.518

For the I1, O2 (framework VI/VII), O3, S, and T superfamilies, the *Dn* and *Ds* values were nearly doubled or tripled from exon1 to exon2 to exon3. Exon1 contains the signal sequence and several bases of the pro-region, which is the most conserved region among the conotoxin genes. Exon2 contains the major pro-region, which is believed to be less conserved than the signal sequence. Exon3 encodes the mature sequence, the region with the greatest diversity among the conotoxin genes. The *Dn* and *Ds* values of exon3 were much higher than those of the other two exons, indicating that many more mutations occurred in exon3.

For the A superfamily, we tested the *Dn* and *Ds* values separately in four subfamilies: α3/5, α4/4, α4/7, and framework IV, according to their different scaffolds (Table 5, Text S1) [26]. Similar to other superfamilies, the A superfamily have an exon1 sequence with a fixed length, and exon2 sequences containing many indels. The gaps among these sequences were deleted in each subfamily to achieve optimal alignment. For the four subfamilies, the *Dn* and *Ds* values of exon2 were 2- to 8-fold greater than those of exon1. It is apparent that many more mutations have occurred in exon2.

In previous studies, the phylogenetic analysis of conotoxins was performed separately according to their signal peptide, propeptide and mature peptide [19], [47]. As a comparison, we also computed the overall mean distance for *Dn* and *Ds* of signal peptide, propeptide and mature peptide within the six conotoxin superfamilies (Table S3). Because of the highly conservation of the signal peptide, the *Dn* and *Ds* values of exon1 and signal peptide are almost the same. In the S superfamily, the *Dn* and *Ds* values of exon2/3 are also very close to those of pro/mature peptide, while in other superfamilies, they have some differences. For the A superfamily, the *Dn* and *Ds* values of propeptide are smaller than those of exon2; while the values of mature peptide are larger than exon2. In our opinion, the results of analyses based on gene structure are likely to be more accurate.

The significant differences in base substitution rates among exons may have occurred because different exons can be synthesized by different DNA polymerases [14], [19]. At present, there are five known families of DNA polymerases [56], [57]. The polymerases in families A, B and C generally contain intrinsic 3′ exonucleolytic proofreading activity, with high fidelity. In contrast, most family X and Y polymerases lack proofreading activity and thus could generate high base substitution and simple indel error rates. In eukaryotes, replication is carried out by *Polγ*, *Polα*, *Polδ* and *Polε*, which are from family A and B, whereas the other polymerases perform various DNA repair tasks. Lower fidelity DNA polymerases usually play an important role in species evolution and in the generation of competent organisms that can survive in changing environments. Polymerases such as *Polζ*, *Polη*, *Rev1* and *Polθ*, all of which lack proofreading activity and have lower nucleotide selectivity, are implicated in the somatic hypermutation (SHM) of immunoglobulin genes [58]. *Polη* preferentially generates T to C and A to G transitions; *Rev1* mainly generates transversion mutations at G/C bases; and *Polζ* could be important in producing all the mutations that occur during SHM. We can also easily find these mutations in conotoxin genes. Thus, we hypothesized that these less accurate polymerases might play a similar role in generating the diversity of conotoxin genes. In conotoxins, the mature peptide exon could be replicated by one or several polymerases from family X or Y, which resulting in the high mutation and indel rates of the mature toxins.

### Positively selected sites are mainly located in the mature peptide exon

A-, I1-, O2- (framework VI/VII), O3-, S-, and T-conotoxin genes were found to have significantly positively selected sites when using the likelihood ratio test (Table 6 and 7) [26]. In the A superfamily, which can be divided into four subfamilies, the positively selected sites were almost all distributed in exon2. Most amino acids in the two loops of the α4/4 and α4/7-subfamilies are positively selected sites (Table 6). In contrast, in the α3/5-subfamily, the three residues in loop1, which may be related to the conotoxin structural stability, are conserved. The five amino acids in loop2 are more diverse; three of them (56K, 58V, 59K) are positively selected sites (Table 6), and they may be related to the function diversity of the α3/5-toxins. For the other superfamilies, the majority of the positively selected sites are located in exon3 (Table 7). In addition, exon1 of the S superfamily and exon2 of the I1 and T superfamilies also contain some positively selected sites (Table 7). This result suggested that the selection pressure was strengthened from exon1 to exon3. There is strong selection on the exon3 (exon2 for A superfamily), which encodes the mature peptide of conotoxins. The selection pressure favours new amino acid substitutions in the mature peptide. The substitutions especially those occurred in the cysteine loops, should be closely related to the functional diversity of conotoxins. While there is strong negative selection on exon1, which could maintain the original sequences of signal peptides, keeping them highly conserved.

**Table 6:** Positively selected sites in A superfamily.

	Exon1		Exon2	
Subfamily	M1/M2	M7/M8	M1/M2	M7/M8
α3/5	-	-	59K*	41K*, 56K*, 58V*, 59K**
α4/4	-	23T*	53H*, 59V**, 60W*	39T**, 40P*, 42L**, 44A*, 45P*, 47I**, 50Y*, 53H**, 54R*, 56P**, 58M**, 59V**, 60W**
α4/7	24S**	-	50G*, 53A**, 54R**, 56A**, 58**, 59G**, 60I**, 64L**	50G**, 53A**, 54R**, 56A**, 58**, 59G**, 60I**, 64L**
Framework IV	-	-	39A**, 40P**, 41W**, 46T**, 58M*, 61P**, 67T**	39A**, 40P**, 41W**, 46T**, 58M**, 61P**, 67T**

α3/5 — Ac1.2, α4/4 — ImIIA, α4/7 — Lt1b, Framework IV — A4.1. *: P>95%; **: P>99%. Amino acids refer to first sequence:

**Table 7:** Positively selected sites in other superfamilies.

	Exon1		Exon2		Exon3	
Superfamily	M1/M2	M7/M8	M1/M2	M7/M8	M1/M2	M7/M8
I1	-	-	20E**, 21K*, 22S**, 24E*, 25R**, 28S*, 29G*, 31A*, 32L**, 33R**, 34G**, 35D**	20E**, 21K**, 22S**, 24E**, 25R**, 28S**, 29G**, 31A**, 32L**, 33R**, 34G**,35D**	41N**, 42K*, 45L**, 48D*, 59V*, 60D*, 61N**, 62K*, 64A**, 65H**, 67I*, 68L**, 69L*, 71N**	41N**, 42K**, 45L**, 47G*, 48D*, 54W*, 59V*, 60D*, 61N**, 62K*, 64A**, 65H**, 66L**, 67I*, 68L**, 69L*, 71N**
O2-VI/VII	21I**	17T*, 21I**, 22Q*	43V**, 46S**	43V**, 46S**	52E**, 56W*, 57S**, 58N**, 59Y**, 62S**, 63H*, 64S**	52E**, 56W**, 57S**, 58N**, 59Y**, 61T*, 62S**, 63H*, 64S**, 70G**, 71E*
O3	16F*	16F*	-	-	68T*, 72P*, 74S**, 77P**, 78Q**	68T*, 72P*, 74S**, 77P**, 78Q**
S	3L*, 16I*, 18P*, 20S**	3L*, 16I*, 18P*, 20S**	42A*	38Y*, 40T*, 42A**	54H**, 66D*, 82F*, 87T*	45T*, 49S*, 54H**, 57E**, 59G*, 62R*, 64T*, 66D**, 68S**, 69G*, 70Y*, 71S*, 74R*, 76G*, 79A**, 81H*, 82F**, 84R**, 87T**
T	-	4F*	24P*, 25K*, 30M**, 31P*, 33A**, 35F*, 36H**	23R*, 24P**, 25K*, 29G*, 30M**, 31P**, 33A**, 35F**, 36H**	54K**, 55H**, 58A**	54K**, 55H**, 58A**

— Tx11.4, O2-VI/VII — lt7a, O3 — ArMSGL-0143, S — S8.1, T— SMRCL-3. *: P>95%; **: P>99%. Amino acids refer to first sequence: I1

### Gene structures and intron sequences have certain impact on the evolution and diversity of conotoxin genes

The genus *Conus* contains a large number of species with relatively small habitats and limited preys. An arms race may occur between *Conus* species and their prey [26]. Thus, cone snails must produce a variety of multi-functional toxins to ensure their survival. At the molecular level, conotoxin genes are often present in multiple copies in the genome (Table S1). Some regulatory elements exist in the intronic sequences of these genes and could influence their expression. Due to the unique gene structures of conotoxins, different evolutionary mechanisms could act on different exons, resulting in significant differences in the base substitution and indel rates and the number of positively selected sites of different exons. Many more point mutations and trinucleotide indels occurred in the mature peptide exon, which drove the molecular diversity of conotoxins, further enhancing their functional diversity. Moreover, rare gene recombination or alternative splicing events can also lead to the generation of new conotoxin genes. In conclusion, the diversity of conotoxin genes could be influenced by their gene structures and intronic sequences.

## Methods

### Ethics statement

The specimens were collected from public water bodies in China, and no specific permissions were required. We have confirmed that our studies did not involve endangered or protected species.

### Specimens

The specimens of Conus textile, Conus caracteristicus, Conus miles, Conus capitaneus, Conus rattus, Conus vitulinus, Conus varius, Conus terebra, Conus betulinus, Conus emaciatus, Conus lividus, Conus quercinus, Conus vexillum, Conus coronatus, Conus ebraeus, Conus tessulatus, Conus litteratus and Conus striatus were collected from reef flats in West Island near Sanya, China. The venom ducts were immediately frozen in liquid nitrogen and then homogenized and resuspended in Trizol reagent (Invitrogen, Carlsbad, USA).

### Preparation of mRNA and genomic DNA

Total RNA was extracted according to the instruction manual provided with the Trizol reagent kit. Pure poly A^+^ mRNA was isolated from total RNA using Oligotex mRNA Kits (QIAGEN, Hilden, Germany), quantified and verified for quality using a RNA 6000 Pico Assay on an Agilent 2100 Bioanalyzer instrument. Genomic DNA was prepared from 30 mg of frozen tissue from each species using the E.Z.N.A. ™ Mollusc DNA Kit (OMEGA, USA) according to the manufacturer's standard protocol.

### cDNA cloning, gDNA PCR and sequencing

The SMARTer PCR cDNA Synthesis Kit (Clontech Laboratories Inc., USA) was used to produce first-strand and second-strand cDNA for 18 *Conus* species. We produced cDNA from 50 ng poly A^+^ mRNA as recommended by the manufacturer except that the 3′ SMART CDS Primer II A and 5′ PCR Primer II A were modified as previously described [59]. To amplify the coding sequences of all 19 conotoxin superfamilies of the 18 *Conus* species, PCR primers were designed to recognize conserved 5′UTR/signal sequence and 3′UTR regions [9], [10], [15], [19], [21], [60], [61], [62].

PCR products were separated using 1.1% agarose gel electrophoresis and purified using the Gel Extraction Kit (OMEGA, USA) according to the manufacturer's standard protocol. Purified PCR products were ligated into the pGEM-T Easy vector (Promega Inc., USA). Ligated vectors were transformed into *E. coli* DH5α competent cells by heat shock. Blue/white colony screening was performed on LB agar plates to select the positive colonies. DNA sequencing was carried out using an ABI PRISM® 3730 automated DNA Analyzer.

Genomic DNA PCR using a pair of primers designed based on the 5′UTR/signal sequence and 3′UTR elements conserved in all 19 superfamilies was performed to obtain the gene sequences of conotoxins within each superfamily. The amplified PCR products were extracted and cloned as described above.

### Genome walking

Based on the results of PCR from the cDNA libraries, *C. textile* (molluscivorous), *C. terebra* (vermivorous) and *C. striatus* (piscivorous), which correspond to the different feeding types of cone snails, were selected for genome walking experiments. The GenomeWalking libraries were constructed using the Universal GenomeWalker™ kit (Clontech Laboratories Inc., USA) according to the manufacturer's instructions. Briefly, the procedure involved the construction of adaptor-ligated libraries (GenomeWalker uncloned libraries) generated by the separate restriction digestion of genomic DNA with *DraI*, *EcoRV*, *PvuII*, and *StuI*, followed by ligation to a special adapter provided in the kit. The “genome walking” involved two sets of primers: adapter primer1 (AP1-sense) 5′-GTAATACGACTCACTATAGGGC-3′ and nested PCR adapter primer2 (AP2-sense) 5′-ACTATAGGGCACGCGTGGT-3′, both provided in the kit, and gene-specific primers designed from the signal peptide regions, the pro-regions and the mature peptide regions of the cDNA sequences of conotoxins from different superfamilies. Primary and nested PCR were performed as recommended by the manufacturer (BD Genome-Walker™). Then, the PCR products were purified, cloned and sequenced as described above.

### Sequence analysis

Gene superfamilies, signal peptides, and cleavage sites of conotoxins were predicted using the ConoPrec tool in ConoServer (http://www.conoserver.org) and the SignalP algorithm (http://www.cbs.dtu.dk/services/SignalP). Identity and similarity assessments were performed using the Basic Local Alignment Search Tool (BLAST, http://blast.ncbi.nlm.nih.gov/Blast.cgi). Nucleotide and amino acid multiple alignments were generated using ClustalX or GENEDOC and refined manually. Synonymous versus nonsynonymous substitution rates were analyzed with MEGA5. PMAL was used for the positive selection test [63]. To detect positive selection sites in each conotoxin superfamily, we tested four models in the program codeml: M1, M2, M7 and M8. Likelihood ratio tests were used to compare M1 with M2 and M7 with M8 using the chi-square test [26]. Bayes Empirical Bayes (BEB) analysis was used to identify positively selected sites [64]. A site was considered positively selected if the posterior probability (pp) was greater than 95%.

### Quantitative real-time PCR

The cDNA and genomic DNA sequences of the A-conotoxin S1.10 precursor were inserted into a modified pFXB-flag vector using *KpnI* and *XbaI*. Both constructs were confirmed by sequencing. One day prior to transfection, HEK 293T cells were seeded in 6-well plates at a concentration of 10^5^ cells/well. The recombinant plasmid DNA was cotransfected with pEGFP-C1 into HEK 293T cells using Lipofectamine 2000 (Invitrogen, USA). Recombinant plasmid DNA was introduced at a concentration of 4 µg/well. At 12, 24, 36 and 48 hours after transfection, total RNA was isolated from the transfected HEK 293T cells using the Trizol kit (Invitrogen, USA). After digestion using the TURBO DNA-*free™* kit (Ambion, USA) to eliminate the genomic contamination, cDNA was synthesized with the ReverTra Ace -α- kit (Toyobo, Japan). pEGFP-C1 was used as an internal standard reference to control for differences in transfection efficiency. After qualification of the cDNA templates and primers, real-time PCR was performed on a LightCycler® 480II instrument (Roche, USA). SYBR Premix® Ex TaqTM (Toyobo, Japan) was used according to the manufacturer's protocol. After PCR, the LightCycler® 480 software was used to analyze the data and calculate the relative mRNA expression level. Each experiment was performed at least in triplicate and repeated three times in all cases.

## Supporting Information

Figure S1 **Sequence alignment of the two introns of 13 conotoxins from nine superfamilies.** The intron sequence of A-conotoxin Vr1.2 was selected to algin with each intron sequence of the other eight superfamilies. Only the first and last 80 bp of these sequences are shown.
(PDF)

Figure S2 **Sequence alignment of 16 introns of the A superfamily.** For the conotoxins that contained the same coding regions, only the longest region has been aligned. Only the first and last 80 bp of these sequences are shown.
(PDF)

Table S1 **Predicted sequences of all A superfamily conotoxins cloned in this paper.**
(PDF)

Table S2 **The Percent Identity Matrix of the A superfamily intron sequences.**
(PDF)

Table S3 ***Dn*** **(top) and** ***Ds*** **(bottom) values of the signal peptide region, propeptide region and mature peptide region within six conotoxin superfamilies.**
(PDF)

Text S1 **All conotoxin gene sequences analyzed.**
(PDF)
