# Correction: Molecular Evolution and Diversity of *Conus* Peptide Toxins, as Revealed by Gene Structure and Intron Sequence Analyses

**DOI:** 10.1371/annotation/98e70cd9-f5d7-4937-bdbc-c68bde86e8cf

**Published:** 2013-12-30

**Authors:** Yun Wu, Lei Wang, Maojun Zhou, Yuwen You, Xiaoyan Zhu, Yuanyuan Qiang, Mengying Qin, Shaonan Luo, Zhenghua Ren, Anlong Xu

The affiliations for the first three authors are incorrect. The first three authors are not affiliated with institution number 2, but rather with institution number 1, State Key Laboratory of Biocontrol, Guangdong Province Key Laboratory of Pharmaceutical Functional Genes, National Engineering Research Center of South China Sea Marine Biotechnology, Department of Biochemistry, College of Life Sciences, Sun Yat-sen University, Guangzhou, China.

