# Supplementary material for: Molecular Evolution and Diversity of Conus Peptide Toxins, as Revealed by Gene Structure and Intron Sequence Analyses
Source: PLoS One. 2013 Dec 13;8(12):e82495. doi: 10.1371/journal.pone.0082495 (PMC3862624; doi:10.1371/journal.pone.0082495)
Supplement: Figure S1 — Sequence alignment of the two introns of 13 conotoxins from nine superfamilies. The intron sequence of A-conotoxin Vr1.2 was selected to algin with each intron sequence of the other eight superfamilies. Only the first and last 80 bp of these sequences are shown. (PDF) [file pone.0082495.s001.pdf]

Intron1-beginning

```
                       *       20         *       40         *       60         *       80
Vr1.2    : GTAAGTCAATATCCGAATTCTCATATGTTGTTACCTGGAGAAGTCTACATTTGTTGCTTTGAACCGGAGTGTAGTGCACT
Tx11.3   : GTAGGTTCCCCTCCCCCCTCTTCTCTCTCTCTCTCTGTCTCTCTCTCTCTGCCTGTCTGTCTCTCTCTCTCTCTCTCT
Tx3.9    : GTGAGATGTTCAGTTTGTTCATCTGTTTCTGTCTGTCTATCTGTCTGACACGCAGATTACTTCTGGTTCATTATCCCTCC
Tx7.29   : GTGTGTGTGTGTGTGTGTTGTGGTGTGTGTGTGTGTGTGTGTGTGTGTGTGTGTGTGTGTGCGTTTGGTCTGTCTGCCGT
Tx15a    : GTGAGATATTTACTTACCTTTCTTCATCTGTTGATCTGTCTATTTGCCCTCTTGTTTGTCTGTCTGCCGTTTATTTATGG
S6.16    : GTACGTGTTGACTGTCCCTGTCTGCTGCCTGGCTGTTGGCCCTCTCACGTTGTTTCTAAGTCTTTACTGTTCCTCCCTGC
Tx9a     : GTAAATCTCTCTGTCTGTCTGTCTGTCTGTCTGTCTGTCTGTCTGTCTGCCTGTCTCTCTAACTCTAACTCTTCTGTCTC
Tx8.1    : GTAAGGTGTTAAATGAATGCCTATCTTTTCGTCTTGAAGATTGTTTCATTAAAACCCACTACCATTCCACCTCTTTGGCA
TeAr193  : GTGAGACAAGTTGTTTTGTCAGTTTAGTTCTCCCCCTTGGCATGGCCAGAGATCCTCTTTGCGCATAACAATGATCTAGG
Tr5.1    : GTGAGACCAGTTGTCTTGTCAGTTTAGTTCTTCCCCTTGGCATGATCAGAGATCCTCTTTGCGCATAATAATGAGCTATG
S10.1    : GTGAGACCAGTTGTCTTGTCAGTCTAGTTCTCCCCCTTGGCATGATCAGAGATTCTCTTTGCGCATAATAATGAGCTATG
Tx5.11   : GTGAGGTCAGTTGTCCTGTTAGCTAGTTCTCCCCCTTGTCATTATCAAAGATCCTCTTTACGCATACTAATGATCTACGA
```

Intron1-ending

```
                       *       20         *       40         *       60         *       80
Vr1.2    : GTGTGTGCGGGCGCGCGCGTGCGTGCGTGTGTGTGTGTGTGTGTGTGTGTGTGTGTGTGTGTGTGTGTGTCTGGGTCCAG
Tx11.3   : GGTGTGAGCACATCACTCAGCACACAATGTATTCGCACAGATGATAAGTTATGATAGTCCTTTTTTCATTCCTGTTTTAG
Tx3.9    : TCTTTTGCTGTCTCTATGTCCCCATAACCACACACATTAATGACAATGCTAAGTGAAGTACCCTGTAATTGATTTTGCAG
Tx7.29   : ACACACACACACACACACACACACACACACACACACACACACACACACACACGTTTAATTAAGTGAATTTGACTTTGCAG
Tx15a    : GATGGTTCTCATGACAGTTAACACATCCACACACGCACGTGCACGCACACGAGCTTAATGAAGTGACTTTGACTTTGCAG
S6.16    : AGCCCTGATATGGCCCTTTGTGGTCGGCTGGGCTATAAGCAACATTATCTTATCTTATCATGTTGTCCACGGCTTTGCAG
Tx9a     : GACAATGATCGATAGAGGCTGCTCATCTATCAGTACCAACGTAAGGTGTACTGAACGATCAGTATCCCTGTTTCTTCCAG
Tx8.1    : TCAGCGTATAGCCTAAGTCTCACAGACATGTCGGAATGGTCTTGAATATTCTTGAGACTGTAACTGTTTCTCTTCTTCAG
TeAr193  : CGTACATATAGGGTCATGAGTGTGTGTGTGTGTGTGTACGTGAGACGTTCACTCGAAATTCTGTCTCGATTTTTCCCCAG
Tr5.1    : GTGTGTGTGTGTGTGTGTGTGTGTGTGTGTGTGTACTTGAGGGGTTCCACTCGAAACTCTGTGTCTCGATTTTTCCCCAG
S10.1    : GCGTGCGTGCGTGTGTGTGTGTGTGAGTGTGTGTACTTGAGGGGTTCCACTCGAAACTCTGTGTCTCGATTTTTCCCCAG
Tx5.11   : GTGTGTGTGTGTGTGTGTGTGTGTGTATGTGCGCACATGAGAGGTTCACTGGAAATTCTATGTCTCGATTTTTCCCCAG
```

Intron2-beginning

```
                        *        20         *        40         *        60         *        80
Vr1.2    : GTAAGTCAATATCCGAATTCTCATATGTTGTTACCTGGAGAAGTCTACATTTGTTGCTTTGAACCGGAGTGTAGTGCACT
Tx11.3   : GTAAGTGCAAAGGACTATGTAACACTCAAAAATACTTTTGATTTAGACAATATCTTAATAAATAATGTATATTCTTCTGT
Tx3.9    : GTAAATCTCTCTCTCTCTCTCTCTCTCTCTCTCTCTCTCTCTCTCTCTCTCTCTCTCTCTCCTCTGTGTGTGTGTGTGTG
Tx7.29   : GTAAGATGTCTATGCTTCTGTTTCTTCCCTTCATCTACCAAAATACCTGTCTCTACTTTTCTAAGACAAATGAAACAAAC
Tx15a    : GTAATATGTAGATTCCTTCCACGCAGTTCCCTTTACTTCCTCATCTGCCTACATGCCTGTCTCTATGTTTCTATTAAACA
S6.16    : GTACGATGTTTTTCATATTCTTCCCTAATCTCTCTCTCTTTCTCTCTCTCTCTCTCTCTGCGGATACGTGTGTGCGCCCC
Tx9a     : GTAGGTATTCTGTCATATTTTCTGTCCATTATCATTTTGAAAAAAAATGGAGATGCCTTTATGTTGTCTCTGCGGGACTT
Tx8.1    : GTACCTGTTTTTCTTATAATCTCCCCCTCCCCTTCCTCCATGTGCGCGTGGGTGACTGTGTGTGTGTGTGTGTGTGTGTG
TeAr193  : GTGGGTGTGTGAGTGAATGTGTGTGTGTGTGTGTGTGTGTGTGTGTGTGTGTGTGTGTGCATGTGTGTGCGCATGTATGTATAT
Tr5.1    : GTGGGTGTTTGTGTGTGTGTGTGTGTGTGTGTGTGTGTGTGTGTGTGTGTGTGTGTGTGTGTGTGTGTAAAATGTGTTTACG
S10.1    : GTGGGTGTGTGAGTGAATGAGAGAGAGAGAGAGAGAGAGAGAGAGAGAGAGAGAGAGAGAGAGAGAGAGAGAGAGAGAGAGA
Tx5.11   : GTGGGTGTGTGAGTGAATGTGTGTGTGTGTGTGTGTGTGTGTGTGTGTGTGTGTGTGTGTGTGTGTGTGTGTGTTTGGGACT
```

Intron2-ending

```
                        *        20         *        40         *        60         *        80
Vr1.2    : GTGTGTGCGGGCGCGCGCGCGTGCGTGCGTGTGTGTGTGTGTGTGTGTGTGTGTGTGTGTGTGTGTGTGTCTGGGTCCAG
Tx11.3   : TCATGATCACTATCATTTAGTCTACCATTTCATCCTTGTTGCTCTTTCACTGCATTTTCTTTCATCGGTATGCTCAACAG
Tx3.9    : AATGCATCATATATCCAGCTTCATCACAACAATTCGCATTGTAATTCCCGGGACTGACGGTTTTGTCACTCTGTTCACAG
Tx7.29   : TGTACTTTTTGTAGATAATGAGCACACTAACATGTTGCAAATAATTTGGTTTGCACATGCTCTTCTATTGTCATTTCCAG
Tx15a    : TGTTCTGTTTCGCAGATCATGGATACACAAATATATTGCAGATAATGAGCTTTTCACATGCACGTTTTTGTCCTTTCCAG
S6.16    : AGGAATACACTGACAATGAATGAAATAGTGTGTTGCTGTTGATCGTCTTCATCCACAGTCTTTCTTTTTCTTCTTCACAG
Tx9a     : CTGTAGTTATCGAGCAGCAGTACGGACATTCTGACACGGTCGTTAGGACAATGATTGTCTCTTTGCTTTTTGTTTTTCAG
Tx8.1    : ACTATGTCGTCCAGCCTCTCCAGCACGTGACGTGTCTCGTCCTCTCCCTCTCTCTCTCTCTCTCTCTCTCTTCGACAG
TeAr193  : CTGTCTGTCTGTGTGTGTCTGTTTGTGTGTGTGTGTGTGTGTGTGTGTGTGTGTGTGTGTGTGTGTGTGTGTGTGCAG
Tr5.1    : GTGCGTGCGTGCGTGCGTGCGTGCGTGTGTGTATATGTGTGTGTGTGTGTGTGTGTGTGTGTGTGTGTGTGTGTGCAG
S10.1    : GTGTGTGTGTGTGTGTGTGTGTGTGTGTGTATGTGTGTGTGTGTGTGTGTGTGTGTGTGTGTGTGTGTGTGTTTGTGCAG
Tx5.11   : CTCTGTGTGTGTGTGTGTGTGTGTGTGCGTGTGTGTGTGTGTGTGTGTGTGTGTGTGTGTGTGTGTGTGTGTGTGCAG
```
