# Supplementary material for: Molecular Evolution and Diversity of Conus Peptide Toxins, as Revealed by Gene Structure and Intron Sequence Analyses
Source: PLoS One. 2013 Dec 13;8(12):e82495. doi: 10.1371/journal.pone.0082495 (PMC3862624; doi:10.1371/journal.pone.0082495)
Supplement: Figure S2 — Sequence alignment of 16 introns of the A superfamily. For the conotoxins that contained the same coding regions, only the longest region has been aligned. Only the first and last 80 bp of these sequences are shown. (PDF) [file pone.0082495.s002.pdf]

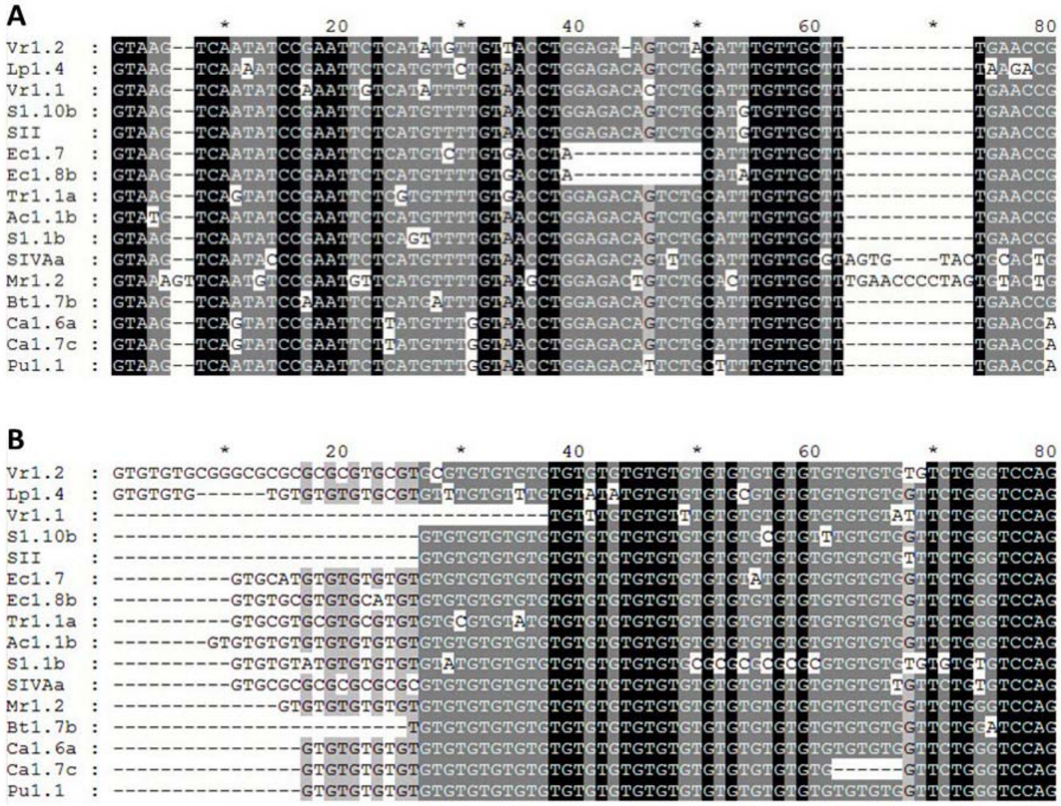
A
B
Vr1.2
Lp1.4
Vr1.1
S1.10b
SII
Ec1.7
Ec1.8b
Tr1.1a
Ac1.1b
S1.1b
SIVAa
Mr1.2
Bt1.7b
Ca1.6a
Ca1.7c
Pu1.1
