# Supplementary material for: Molecular Evolution and Diversity of Conus Peptide Toxins, as Revealed by Gene Structure and Intron Sequence Analyses
Source: PLoS One. 2013 Dec 13;8(12):e82495. doi: 10.1371/journal.pone.0082495 (PMC3862624; doi:10.1371/journal.pone.0082495)
Supplement: Table S1 — Predicted sequences of all A superfamily conotoxins cloned in this paper. (PDF) [file pone.0082495.s003.pdf]

Table S1: Predicted sequences of all A superfamily conotoxins cloned in this paper.

Conotoxin	*Conus* species	Introns (bp)	Mature toxin	Branch	GenBank number
Vr1.1	*C. varius*	1262	ATWDYCCPPYICDTHFPSHCK	α4/7	JX293552
Vr1.2	*C. varius*	1422	AIGGTCCPIPSCASTFPRLCG	α4/7	JX293551
Tr1.1a	*C. terebra*	1068	DCCSNPPCAHNNPDCR	α4/6	JX293550
Tr1.1b	*C. terebra*	1044	DCCSNPPCTHNNPDCR	α4/6	JX293549
Tr1.1c	*C. terebra*	1038	DCCSNPPCSHNNPDCR	α4/6	JX293548
Ec1.7	*C. emaciatus*	1000	DCCSNPPCAHNIHCA	α4/5	JX293547
Ec1.8a	*C. emaciatus*	993	DCCSDPPCAHNNPDCR	α4/6	JX293546
Ec1.8b	*C. emaciatus*	1056	DCCSDPPCAHNNPDCR	α4/6	JX293545
Ca1.6a	*C. caracteristicus*	1173	YLGIDCCRIPSCFAKYGSKCSKIR	α4/7	JX293544
Ca1.6b	*C. caracteristicus*	1169	YLGIDCCRIPSCFAKYGSKCSKIR	α4/7	JX293543
Ca1.7a	*C. caracteristicus*	1159	SFTINCCKIPSCFAKYGSKCSEVH	α4/7	JX293542
Ca1.7b	*C. caracteristicus*	1156	SFTINCCKIPSCFAKYGSKCSEVH	α4/7	JX293541
Ca1.7c	*C. caracteristicus*	1172	SFTINCCKIPSCFAKYGSKCSEVH	α4/7	JX293540
Bt1.7a	*C. betulinus*	970	GGCCSYPACSVEHQDLCD	α4/7	JX293539
Bt1.7b	*C. betulinus*	996	GGCCSYPACSVEHQDLCD	α4/7	JX293538

Bt1.7c	*C. betulinus*	990	GGCCSYPACSVEHQDLCD	α4/7	JX293537
Bt1.7d	*C. betulinus*	974	GGCCSYPACSVEHQDLCD	α4/7	JX293536
S1.1a	*C. striatus*	1157	NGCCRNPACESHRCG	α4/4	JX293534
S1.1b	*C. striatus*	1259	NGCCRNPACESHRCG	α4/4	JX293533
S1.10a	*C. striatus*	1119	AYCCHPACGKNFDCGR	α3/5	JX293554
S1.10b	*C. striatus*	1133	AYCCHPACGKNFDCGR	α3/5	JX293553
SII	*C. striatus*	1159	GCCCNPACGPNYGCGTSCSRTI	Framework II	JX293535
SIVAa	*C. striatus*	1327	QKSLVPSVITTCCGYDPGTMCPPCRCTNSCG	Framework IV	JX293532
SIVAb	*C. striatus*	1260	QKSLVPSVITTCCGYDPGTMCPPCRCTNSCG	Framework IV	JX293531
