# Supplementary material for: Molecular Evolution and Diversity of Conus Peptide Toxins, as Revealed by Gene Structure and Intron Sequence Analyses
Source: PLoS One. 2013 Dec 13;8(12):e82495. doi: 10.1371/journal.pone.0082495 (PMC3862624; doi:10.1371/journal.pone.0082495)
Supplement: Table S2 — The Percent Identity Matrix of the A superfamily intron sequences. (PDF) [file pone.0082495.s004.pdf]

Table S2: The Percent Identity Matrix of the A superfamily intron sequences.

	Conotoxin															
Conotoxin	Vr1.1	Vr1.2	S1.10b	SII	Ac1.1b	S1.1b	SIVAa	Ec1.7	Ec1.8b	Tr1.1a	Bt1.7b	Mr1.2	Ca1.6a	Ca1.7c	Pu1.1	Lp1.4
Vr1.1		86	79	79	78	79	75	78	78	77	63	69	50	49	49	85
Vr1.2			78	77	76	76	73	77	76	75	63	69	51	51	51	82
S1.10b				100	92	92	84	86	86	86	64	70	54	54	54	75
SII					92	91	83	86	86	86	65	70	54	53	53	75
Ac1.1b						88	82	86	86	85	64	71	53	53	52	72
S1.1b							84	84	84	84	63	70	53	52	52	75
SIVAa								79	78	79	63	66	51	50	50	68
Ec1.7									98	95	65	69	53	52	52	67
Ec1.8b										95	65	69	52	52	52	67
Tr1.1a											66	69	54	53	53	67
Bt1.7b												68	51	50	50	51
Mr1.2													56	55	55	61
Ca1.6a														99	96	54
Ca1.7c															95	53
Pu1.1																52
Lp1.4																
