# Supplementary material for: Molecular Evolution and Diversity of Conus Peptide Toxins, as Revealed by Gene Structure and Intron Sequence Analyses
Source: PLoS One. 2013 Dec 13;8(12):e82495. doi: 10.1371/journal.pone.0082495 (PMC3862624; doi:10.1371/journal.pone.0082495)
Supplement: Table S3 — Dn (top) and Ds (bottom) values of the signal peptide region, propeptide region and mature peptide region within six conotoxin superfamilies. (PDF) [file pone.0082495.s005.pdf]

Table S3. *Dn* (top) and *Ds* (bottom) values of the signal peptide region, propeptide region and mature peptide region within six conotoxin superfamilies.

Region	Superfamily								
	A				I1	O2-VI/VII	O3	S	T
	α3/5	α4/4	α4/7	IV					
Signal peptide	0.034<br>0.027	0.057<br>0.091	0.062<br>0.108	0.021<br>0.008	0.061<br>0.118	0.058<br>0.094	0.057<br>0.068	0.044<br>0.000	0.083<br>0.141
Propeptide	0.086<br>0.031	0.391<br>0.159	0.257<br>0.197	0.057<br>0.016	0.201<br>0.077	0.274<br>0.256	0.111<br>0.078	0.189<br>0.131	0.258<br>0.211
Mature peptide	0.120<br>0.081	0.534<br>0.422	0.471<br>0.469	0.246<br>0.110	0.616<br>0.748	0.497<br>0.639	0.339<br>0.286	0.509<br>0.394	0.467<br>0.786
