# Supplementary material for: Molecular Evolution and Diversity of Conus Peptide Toxins, as Revealed by Gene Structure and Intron Sequence Analyses
Source: PLoS One. 2013 Dec 13;8(12):e82495. doi: 10.1371/journal.pone.0082495 (PMC3862624; doi:10.1371/journal.pone.0082495)
Supplement: Text S1 — All conotoxin gene sequences analyzed. (PDF) [file pone.0082495.s006.pdf]

All the conotoxin gene sequences for analysis.

A superfamily

alpha3/5 family

>Ac1.2 GenBank number: BD261435.1
ATGTTCACCGTGTTTCTGTTGGTTGTCTTGACAACCACTGTCGTTTCCTTCCCTTCAGATAGTGCATCTG
GTGGCAGGGATGACGAGGCCAAAGACGAAAGGTCTGACATGTACGAATTGAAACGGAATGGACGC----
--TGTTGCCATCCTGCCTGTGGTGGCAAATACGTTAAATGTGGACGC

>MnII GenBank number: BD394971.1
ATGTTCACCGTGTTTCTGTTGGTTGTCTTGACAACCACTGTCGTTTCCTTCCCTTCAGATAGTGCATCTG
GTGGCAGGGATGACGAGGCCAAAGACGAAAGGTCTGACATGTACGAATTGAAACGGAATGGACAC----
--TGTTGCCATCCTGCCTGTGGTGGCAAATACGTTAAATGTGGACGC

>Cn1.3 GenBank number: FJ868076
ATGTTCACCGTGTTTCTGTTGGTTGTCTTGACAACCACTGTCGTTTCCTTCCCTTCAGATCGTGCATCTG
ATGGCAGGGATGACGAAGCCAAAGACGAAAGGTCTGACATGTACAAATCGAAACGGAATGGACGC----
--TGTTGCCATCCTGCCTGTGGTGGCAAATACTTTAAATGTGGACGC

>Ac1.1b GenBank number: DQ311072.1
ATGTTCACCCTGTTTCTGTTGGTTGTCTTGACAACCACTGTCGTTTCCTTCCCTTCAGATAGTGCATCTG
ATGGCAGGGATGACGAAGCCAAAGACGAAAGGTCTGACATGTACAAATCGAAACGGAATGGACGC----
--TGTTGCCATCCTGCCTGTGGC---AAACACTTTAGTTGTGGACGC

>MnI GenBank number: BD394976.1
ATGTTCACCGTGTTTCTGTTGGTTGTCTTGACAACAACTGTCGTTTCCTACCCTTCAGATAGTGCATCTG
ATGGCAGGGATGACGAAGCCAAAGACGAAAGGTCTGACATGTACAAATCGAAACGGAATGGACGC----
--TGTTGCCATCCTGCCTGTGGC---AAACACTTTAGTTGTGGACGC

>Cn1.1 GenBank number: BD261416.1
ATGTTCACCGTGTTTCTGTTGGTTGTCTTGACAACCACTGTCGTTTCCTTCCCTTCAGATAGTGCATCTG
ATGTCAGGGATGACGAAGCCAAAGACGAAAGGTCTGACATGTACAAATCGAAACGGAATGGACGC----
--TGTTGCCATCCTGCCTGTGGC---AAACACTTTAGTTGTGGACGC

>C1 GenBank number: FJ868069
ATGTTCACCGTGTTTCTGTTGGTTGTCTTGACAATCACTGTCGTTTCCTTCCCTTCAGATCGTGCATCTG
ATGGCAGGGATGACGAAGCCAAAGACGAAAGGTCTGACATGTACAAATCGAAACGGAATGGACGC----
--TGTTGCCATCCTGCCTGTGGC---AAACACTTTAGTTGTGGACGC

>M1.1 GenBank number: BD394984.1
ATGTTCACCGTGTTTCTGTTGGTTGTCTTGACAACCACTGTCGTTTCCTTCCCTTCAGATCGTGCATCTG
ATGGCAGGGATGACGAAGCCAAAGACGAAAGGTCTGACATGTACGAATCGAAACGGGATGGACGC----
--TGTTGCCATCCTGCCTGTGGG---CAAAACTATAGTTGTGGACGC

>Cr1.1 GenBank number: BD394977.1
ATGTTCACCGTGTTTCTGTTGGTTGTCTTGGCAGCCACTGTCGTTTCCTTCCCTTCAGATCGTGCATCTG
ATGGCAGGGATGACGAAGCCAAAGACGAAAGATCTGACATGCACGAATCGGACCGGAAAGGACGCG
GACGCTGTTGCCATCCTGCCTGTGGC---CCAAATTATAGTTGTGGACGC

>Sm1.1 GenBank number: BD394966.1
ATGTTCACCGTGTTTCTGTTGGTTGTCTTGGCAACCACTGTCGTTTCCTTCCCTTCAGATCGTGCATCTG
ATGGCAGGGATGACGAAGCCAAAGACGAAAGGTCTGACATGCACGAATCGGGCCGGAAAGGACGCG
GACGCTGTTGCCATCCTGCCTGTGGC---CCAAACTATAGTTGTGGACGC

>S1.9 GenBank number: FJ868105
ATGTTCACCGTGTTTCTGTTGGTTGTCTTGGCAACCACTGTCGTTTCCTTCACTTCAGATCGTGCATCTG
ATGGCAGGGATGACGAAGCCAAAGACGAAAGGTCTGACATGCACGAATCGGACCGGAAAGGACGCG
CATACTGTTGCCATCCTGTCTGTGGC---AAAAACTTTGATTGTGGACGC
>S1.10 GenBank number: FJ868100
ATGTTCATTGTGTTTCTGTTGGTTGTCTTGGCAACCACTGTCGTTTCCTTCCCTTCAGATCATGCATCTG
ATGGCAGGGATGACGAAGCCAAAGAC------GTCGACATGCACGAATCGGACCGGAAAGGACGCGCATA
CTGTTGCCATCCTGCCTGTGGC---AAAAACTTTGATTGTGGACGC

alpha4/4 family
>ImIIA GenBank number: AF200595.1
ATGGGCATGCGGATGATGTTCACCGTGTTTCTGTTGGTTGTCTTGGCAACTGCTGTTCTTCCAGTCACT
TTAGATCGTGCATCTGATGGAAGGAATGCAGCAGCCAACGCCAAAACGCCTCGCCTGATCGCGCCATT
CATCAGGGATTATTGCTGTCATAGAGGTCCCTGTATGGTATGG---TGTGGT
>Qc1.7 GenBank number: JX293446
ATGGGCATGCGGATGATGTTCACCGTGTTTCTGTTGGTTGTCTTGGCAACCGCTGTCGTTTCCTTCAGT
TCAGATCGTGCATCTCATGGCAGGAATGCCGCAGCC------AAAGTGTTTGACCGGATCGCTCTGATCGC
CACACGCGAATGCTGTGCCAATCCTCAATGTTGGGCGAAGAATTGTCGC
>Eb1.3 GenBank number: JX293432
ATGGGCATGCGGATGATGTTCACCGTGTTTCTGTTGGTTGTCTTGGCAACCGCTGTCGTTTCCTTCAGT
TCAGATCGTGCATCTCATGGCAGGAATGCCGCAGCC------AAATTGTTTGACCGGATCGCTCTGATCGCC
ACACGCGAATGCTGTGCCAATCCTCAATGTTGGGCGAAGAATTGTCGT
>Co1.3 GenBank number: JX293412
ATGGGCATGCGGATGATGTTCACCGTGTTTCTGTTGGTTGTCTTGGCAACCTCTGTCGTTTCCTTCAATT
CAGATCGTGCATCTCATGGCAGGAATGCTGCAGCC------AAAGTGTTTAAGCGGATCGCTCTGATCGCC
ACACGCGAATGCTGTGCCAATCCTCAATGTTGGTCGAAGAATTGTCGT
>Lv1.3 GenBank number: JX293418
ATGGGCATGCGGATGATGTTCACCGTGTTTCTGTTGGTTGTCCTGGCAACCGCTGTCGTTTCCTTCACT
TTAGATCGTGCATCAGATGGCGGGAATGCCGCAGCC------AAAGCGTTTGACCTGATCTCTCTGATCGCC
AGAGAAGATTGTTGTGCCTTGCATGCCTGTTGGATGTAT---TGTCGT
>Lv1.12 GenBank number: JX293423
ATGGGCATGCGGATGATGTTCACCGTGTTTCTGTTGGTTGTCTTGGCAACCACTGTCGTTTCCTTCACT
TCAGATCGTGCATCTCATGCCAGGAATGCCGCAGCC------AAAGTGTTTGACCTGATCTCTCTGATCGCC
AGAGAAGATTGTTGTGCCTTGCATGCCTGTTGGATGTAT---TGTCGT
>Lv1.11 GenBank number: JX293422
ATGGGCATGCGGATGATGTTCACCGTGTTTCTGTTGATTGTCTTGGCAACCACTGTCGATTCCTTCACTT
CAGATCGTGCATCAGATGGCGGGAATGCCGCAGCC------AAAGCGTTTGACCTGATGGCTCTGACCGCC
AGACTAGATTGCTGTGACATTCATGCCTGTTGGATGTAT---TGTCGT
>Qc1.2 GenBank number: AY580320.1
ATGGGCATGCGGATGATGTTCACTGTGTTTCTGTTGGTTGCCTTGGCAACCACTGTCGCTTCCTTCACT
TTAGATCGTGCATCTAATGGCAGGAATGCTGCAGCCGACGACAAACCGTCTGACTGGATCGCTCTGGC
CATCAAG---CAATGCTGCGCCAATCCTCCCTGTAAACATGTAAATTGTCGT
>S1.1 GenBank number: BD261403.1
--------------ATGTTCACTGTGTTTCTGTTGGTTGTCTTGGCAATCACTGTCGTTTCCTTCCCTTTAGATCGT

GAATCTGATGGCGCGAATGCCGAAGCCCGCACCCACGATCATGAGAAGCACGCACTG---GACCGGAAT
GGATGCTGTAGGAATCCTGCCTGTGAGAGCCACAGATGTGGT

>Cr1.3 GenBank number: BD261427.1

--------------ATGTTCACCGTGTTTCTGTTGGTTGTCTTGGCAACCACTGTCGTTTCCTTCCCTTCAAATCG
TGAATCTGATGGCGCGAATGCCGAAGTCCGCACCGACGAGCCTGAGGAGCACGACGAACTGGGCGG
GAATGGATGCTGTGGGAATCCTGACTGTACGAGCCACAGTTGTGAT

>Sm1.3 GenBank number: BD261425.1

--------------ATGTTCACCGTGTTTCTGTTGGTTGTCTTGGCAACCACTGTCGTTTCCTTCCCTTCAGATCG
TGAATCTGATGGCGCGAATGACGAAGCCCGCACCGACGAGCCTGAGGAGCACGGACCG---GACAGGA
ATGGATGCTGTAGGAATCCTGCCTGTGAGAGCCACAGATGTGGT

alpha4/7 family

>Lt1b GenBank number: DQ345365.1

ATGGGCATGCGGATGATGTTCATCATGTTTATGTTGGTTGTCTTGGCAACCACTGTCGATACCTTCACTT
CAGATCGTGCACTTGATGCCATGAATGCTGCAGCCAGCAACAAAGCGTCTCGCCTGATCGCCCTGGCC
GTCAGG---GGATGCTGTGCCCGTGCCGCCTGTGCCGGGATCCATCAAGAACTTTGTGGAGGA------------
CGACGC------------------------------

>Lt1A GenBank number: DQ345364.1

ATGGGCATGCGGATGATGTTCATCATGTTTATGTTGGTTGTCTTGGCAACCACTGTCGTTACCTTCACTT
CAGATCGTGCACTTGATGCCATGAATGCTGCAGCCAGCAACAAAGCGTCTCGCCTGATCGCCCTGGCC
GTCAGG---GGATGCTGTGCCCGTGCTGCCTGTGCCGGGATTCATCAAGAACTTTGTGGAGGA------------
GGACGC------------------------------

>Lp1.1 GenBank number: DQ359141.1

ATGGGCATGCGGATGATGTTCATCATGTTTATGTTGGTTGTCTTGGCAACCACTGTCGTTACCTTCACTT
CAGATCGTGCACTTGATGCCATGAATGCTGCAGCCAGCAACAAAGCGTCTCGCCTGATCGCCCTGGCC
GTCAGG---GGATGCTGTGCCCGTGCTGCCTGTGCCGGGATTCATCAAGAACTTTGTGGAGGA------------
GGACGC------------------------------

>Lp1.4 GenBank number: DQ311056.1

ATGGGCATGCGGATGATGTTCATCATGTTTATGTTGGTTGTCTTGGCAACGACTGTCGTTTCCTTCACTT
CAGATCGTGCACTTGATGCCATGAATGCTGCAGCCAGCAAGAAAGCGTCTCGCCTGATCGCTCTGGCC
GTCAGG---GGATGCTGTTCCCATCCTGCCTGTAGTGGAAATCATCAAGAACTTTGTGATGGA------------A
GACGC------------------------------

>Co1.1 GenBank number: JX293410

ATGGGCATGCGGATGATGTCCACCGTGTTTCTGTTGGTTGTCCTGGCAACCGCTGTCGTTTCCTTCACT
TTAGATCGTGCATCAGATGGCGGGGATGCCGCAGCC------AAAGCGTTTGACCTGATGGCTCTGACCGC
CAGACTAGATTGCTGTGACATTCATGCCTGTTGGTCGGCGCATCCAGAACTTTGTGGAGGA------------A
GACGC------------------------------

>Lv1.2 GenBank number: JX293417

ATGGGCATGCGGATGATGTCCACCGTGTTTCTGTTGGTTGTCCTGGCAACCGCTGTCGTTTCCTTCACT
TTAGATCGTGCATCAGATGGCGGGAATGCCGCAGCC------AAAGCGTTTGACCTGATGGCTCTGACCGC
CAGACTAGATTGCTGTGACATTCATGCCTGTTGGTCGGCGCATCCAGAACTTTGTGGAGGA------------A
GACGC------------------------------

>Ec1.1 GenBank number: JX293436

ATGGGCATGCGGATGATGTTCACCGTGTTTCTGTTGGTTGTCCTGGCAACCGCTGTCGTTTCCTTCACT

TTAGATCGTCCATCAGATGGCGGGGATGCCGCAGCC------AAAGCGTTTGACCTGATGGCTCTGACCGC
CAGACTAGATTGCTGTGACATTCATGCCTGTTGGTCGGCGCATCCAGAACTTTGTGGAGGA------------A
GACGC------------------------------

>Co1.2 GenBank number: JX293411

ATGGGCATGCGGATGATGTTCACCGTGTTTCTGTTGGTTGTCTTGGCAACCGCTGCCGTTTCCTTCACT
TTAGATCGTGCATCAGATGGCGGGAATGCCGCAGCC------AAAGCGTTTGACCTGATGGCTCTGACCGC
CAGACTAGATTGCTGTGACATTCATGCCTGTTGGTCGGCGCATCCAGAACTTTGTGGTGGA------------A
GACGC------------------------------

>Co1.7 GenBank number: JX293416

ATGGGCATGCGGATGATGTTCACCGTGTTTCTGTTGGTTGTCTTGGCAACCGCTGCCGTTTCCTTCACT
TCAGATCGTGCATATGATGGCGGGAATGACGCAGCC------AAAGCGTTTGACTGGATCGCTCTGACCGC
CAGACACGAATGCTGTTCCTATCCTGCCTGTGCGTATACTCATCCAGAACTTTGTGGTGGA------------AG
ACGC------------------------------

>Eb1.6 GenBank number: JX293433

ATGGGCATGCGGATGATGTTCACCATGTTTCTGTTGGTTGTCTTGGCAATTGCTGTCGTTTCCTTCACTT
CAGATCGTGCATCTGATGGCAGGAATGACGCAGCC------AAAGCGTTTCACCGGATCGCTCTGACCGCC
AGAACAGGATGCTGTGAATTTCCTTACTGTGCAGAAAATATTCCAGAATTATGTGGAGGA------------AG
ACGC------------------------------

>Lv1.13 GenBank number: JX293424

ATGGGCATGCGGATGATGTTCACCATGTTTCTGTTGGTTGTCTTGGCAATTGCTGTCGTTTCCTTCACTT
CAGATCGTGCATCTGATGGCAGGAATGACGCAGCC------AAAGCGTTTCACCGGATCGCACTGACCGCC
AGAACAGGATGCTGTGAATTTCCTTACTGTGCAGAAAATATTCCAGAATTATGTGGAGGA------------AG
ACGC------------------------------

>Lp1.3 GenBank number: AY580323.1

ATGGGCATGCGGATGATGTTCATCATGTTTATGCTGGTTGTCTTGGCAACCACTGTCGTTTCCTTCACTT
CAGATCGTGCATCTGATGGCAGGAATGCCGAAGCC------AAAGCGCTTGACTTGATCGCTGCGACCGCC
AGGCCAAGATGCTGTTCCAATCCTGCCTGTGGGGCAGGACATCCGGAAATTTGTGCTGGA------------AG
ACGC------------------------------

>Mi1.1 GenBank number: AY588975.1

ATGGGCATGCGGATGATGTTCACCGTGTTTCTGTTGGTTGTCTTGGCAACCACTGTCGTTTCCTTCACT
TCAGATCGTGGATCTGATGGCAGGAATGCCGCAGCCAAGGACAAAGCGTCTGACCTGGTCGCTCTGA
CCGTCAAG---GGATGCTGTTCTAATCCTCCCTGTTACGCGAATAATCAAGCCTATTGTAATGGA------------
AGACGC------------------------------

>Mr1.2 GenBank number: DQ359140.1

ATGGGCATGCGGATGATGTTCACCGTGTTTCTGTTGGTTGTCTTGGCAACCACTGTCGTTTCCTTCACT
TCAGATCGTGGATCTGATGGCAGGAATGCCGCAGCCAAGGACAAAGCGTCTGACCTGGTCGCTCTGA
CCGTCAAG---GGATGCTGTTCTAATCCTCCCTGTTACGCGAATAATCAAGCCTATTGTAATGGA------------
AGACGC------------------------------

>Ca1.5 GenBank number: JX293428

ATGGGCATGCGGATGATGTTCACCGTGTTTCTGTTGGTTGTCTTGGCAACCACTGTGGTTTCCTTCACT
TCAGATCGTACATCTGATAGCAGGAATGCCGCAGCC------AACGCGTTTGACCTGATCGCTCGGTACCTC
GGGATAGATTGCTGTAGGATTCCCAGCTGTTTTGCGAAATATGGATCCAAATGTAGTAAA------------ATA
CGC------------------------------

>Ca1.4 GenBank number: JX293427

ATGGGCATGCGGATGATGTTCACCGTGTTTCTGTTGGTTGTCTTGGCAACCACTGTGCTTTCCTTCACT
TCAGATCGTGCATCTGAAGGCAGGAATGCTGCAGCCAAGGACAAAGCGTCTGACCTGGTGGCTCTGA
GAGTCAGG---GGATGCTGTGCCATTCATGAATGTCGCTTGCAGAATGCAGCGTATTGTGGTGGA----------
--ATATAC-------------------------------

>Mr1.1 GenBank number: AY580325.1
ATGGGCATGCGGATGATGTTCACCGTGTTTCTGTTGGTTGTCTTGGCAACCACTGTCGTTTCCTTCACT
TCAGATCGTGCATCTGATGGCAGGAAAGCCGCAGCCAAGGACAAAGCGTCTGACCTGGTCGCTCTGA
CCGTCAAG---GGATGCTGTTCTCATCCTGCCTGTAGCGTGAATAATCCAGACATTTGTGGT-------------------
----

>PnIB GenBank number: AF215088.1
ATGGGCATGCGGATGATGTTCACCGTGTTTCTGTTGGTTGTCTTGGCAACCACCGTCGTTTCCTTCACT
TCAGATCGTGCATCTGATGACGGGAATGCCGCAGCA------------TCTGACCTGATCGCTCTGACCATCAA
G---GGATGCTGTTCTCTTCCTCCCTGTGCCCTGAGTAATCCAGACTATTGTGGT---------------------------------
----

>PnMGMR-02 GenBank number: AF215089.1
ATGGGCATGCGGATGATGTTCACCGTGTTTCTGTTGGTTGTCTTGGCAACCACCGTCGTTTCCTTCACT
TCAGATCGTGCATCTGATGGCGGGAATGCCGCAGCG------------TCTGACCTGATCGCTCTGACCATCAA
G---GGATGCTGTTCTCGTCCTCCCTGTGCCCTGAGTAATCCAGACTATTGTGGT---------------------------------
----

>Vc1.3 GenBank number: GU046309.1
ATGGGCATGCGGATGATGTTCACCGTGTTTCTGTTGGTTGTCTTGGCAACCACCGTCGTTTCCTTCACT
TCAGATCGTGCATCTGATGGCAGGAAAGCCGCTGCG------------TCTGACCTGATCACTCTGACCATCAA
G---GGATGCTGTTCTGATCCTCCCTGTATCGCGAATAATCCAGACTTGTGTGGTCGA------------CGACGT--
----

>Tx1.3 GenBank number: JX293425.1
ATGGGCATGCGGATGATGTTCACTGTGTTTCTGTTGGTTGTCTTGGCAACCGCCGTCGTTTCCTTCACT
TCAGATCGTGCATCTGATGACAGGAAAGCCGCTGCG------------TCTGACCTGATCACTCTGACCATCAA
G---GGATGCTGTTCGCGTCCTCCCTGTATCGCGAATGATCCAGACTTGTGTGGT---------------------------------
----

>Vc1.2 GenBank number: GU046308.1
ATGGGCATGCGGATGATGTTCACCGTGTTTCTGTTGGTTGTCTTGGCAACCACCGTCGTTTTCTTCACT
TCAGATCGTGCATCTGATGGCAGGAAAGCCGCTGCG------------TCTGACCTGATCACTCTGACCATCAA
G---GGATGCTGTTCTAATCCTGCCTGTATGGTGAATAATCCACAGATTTGTGGTCGA------------CGACGC--
----

>Lp1.2 GenBank number: AY580322.1
ATGGGCATGCGGATGATGTTCACCGTGTTTCTATTGGTTGTCTTGGCAACCACTGTCGTTTCCTTCACTT
CAGATCGTGCATTTGATGGCAGGAATGCTGCAGCCAGCGACAAAGCGTCTGACCTGATCTCTCTGGCC
GTCAGG---GGATGCTGTTCCCATCCTGCCTGTAGTGTGAATAATCCATACTTTTGTGGTGGA------------AA
ACGC-------------------------------

>Qc1.1a GenBank number: DQ311060.1
ATGGGCATGCGGATGATGTTCACCATGTTTCTGTTGGTTGTCTTGGCAATCACGGTGGTTTCCTTCACC
TCAGATCATGCATCTGATGGCAGGAATACCGCAGCTAACGACAAAGCGTCTAACCTGATGGCTCTTAG
GGAC------GAATGCTGTCCCGATCCTCCGTGTAAGGCGAGTAATCCAGATTTGTGTGACTGG------------A
GAAGC-------------------------------

>Qc1.13 GenBank number: JX293450
ATGGGCATGCGGATGATGTTCACCATGTTTCTGTTGGTTGTCTTGGCAATCACGGTGGTTTCCTTCACC
TCAGATCATGCATCTGATGGCAGGAATACCGCAGCTAACGACAAAGCGTCTAACCTGATGGCTCTTAG
GGAC------GAATGCTGTCCCAATCCTCCGTGTAAGGCGAGTAATCCAGATTTGTGTGACTGG------------A
GAAGC------------------------------
>Qc1.1b GenBank number: DQ311061.1
ATGGGCATGCGGATGATGTTCACCATGTTTCTGTTGGTTGTCTTGGCAATCACGGTGGTTTCCTTCACC
TCAGATCATGCATCTGATGGCAGGAATACCGCAGCCAACGACAAAGCGTCTAAACTGATGGCTCTTAG
GAAC------GAATGCTGTGACAATCCTCCGTGCAAGTCGAGTAATCCAGATTTGTGTGACTGG------------A
GAAGC------------------------------
>Qc1.10 GenBank number: JX293448
ATGGGCATGCGGATGATGTTCACCATGTTTCTGTTGGTTGTCTTGGCAATCACGGTGGTTTCCTTCACC
TCAGATCATGCATCTGATGGCAGGAATACCGCAGCCAACGACAAAGCGTCTAAACTGATGGCTCTTAG
GAAC------GAATGCTGTGACAATCCTCCGTGCAAGTCGAGTAATCCAGATTTGTGTGACTGG------------A
GAAGC------------------------------
>Qc1.9 GenBank number: JX293447
ATGGGCATGCGGATGATGTTCACCATGTTTCTGTTGGTTGTCTTGGCAACCACTGTGGTTTCCTTCACC
TCAGATCATGCATCTGATGGCAGGAATACCGCAGCCAATGACAAAGCGTCTGACCTGATGGCTCTTAG
GGAT------GGATGCTGTTCCAGTCCTTCCTGTTCCGTGAACAATCCAGACATCTGTGGCGGA------------G
GACGC------------------------------
>Eb1.1 GenBank number: JX293430
ATGGGCATGCGGATGATGTTCACCATGTTTCTGTTGGTTGTCTTGGCAACCACTGTGGTTTCCTTCACC
TCAGATCATGCATCTGATGGCAGGAATACCGCAGCCAATGACAAAGCGTCTGACCTGATGGCTCTTAG
GGAT------GGATGCTGTTCCAGTCCTTCCTGTTCCGTGAACAATCCAGACATCTGTGGCGGA------------G
GACGC------------------------------
>Rt1.1 GenBank number: JX293443
ATGGGCATGCGGATGATGTTCACCATGTTTGTGTTGGTTGTCTTGGCAACCACTGTGGTTTCCTTCCCC
TTAGATCATGCATCTAATGGCAGGGATGCCGCAGCCTACGACAAAGCGACTCCCCTGATCGCTCGGGA
CATGGAG---GAATGCTGTTCCCATCCTGCCTGTCAAGGGATTAATCCAGACTATTGTGAT-------------------
----
>Ca1.3 GenBank number: JX293426
ATGGGCATGCGGATGATGTTCACCGTGTTTCTGTTGGTTGCCTTGGCAACCACTGTCGTTCCCTTCACT
TCAGATCGTGCATCTGCTAGCAGGAATGCCGCAACCGACAACAAAGCATCTGAGCTGAAAGCTCTGA
ACGCCAGGATACCATGCTGTTCCTATCCTGCTTGTGCCCAGAGTAATATAGACCTTTGTGGTGGA---------
---AGACGC------------------------------
>Lv1.4 GenBank number: JX293419
ATGGGCATGCGGATGATGTTCACCATGCTTCTGTTGGTTGTCTTGGCAACCACTGTCGTTTCCTTCACG
TTAGATCATGCATTTGATGGCAGGAATACCGCAGCCAACAACAAAGCGACTGACCTGATGGCTCTGCC
TGTCAGG---GGATGCTGTTCCGATCCTCCCTGTAGACACAAGCACCAAGATCTTTGTGGC-----------------
----
>Lp1.7 GenBank number: DQ311062.1
ATGGGCATGCGGATGATGTTCACCATGTTTCTGTTGGTTGTTTTGACAACCACTGTGGTTTCCTTCAATT
CAGATCGTGAATCCAATCACGAGAATCGCAGAACA------------TCTAACCAGATTACGCGGGGCATGTGG
GATGAATGCTGTGACGATCCTCCATGTCGGCAAAATAATATGGAGCATTGTCCCGCAAGT------------------

----
>Lp1.8 GenBank number: DQ311063.1
ATGGGCATGCGGATGATGTTCACCATGTTTCTGTTGGTTGTTTTGACAACTACTGTGGTTTCCTTCAATT
CAGATCGTGAATCCAATCACGAGAATCGCAGAACA------------TCTAACCAGATTACGCGGGGCGTTTGG
GATGAATGCTGTAAAGATCCTCAATGTCGGCAAAATCATATGCAGCATTGTCCCGCACGT------------------
----
>Lt1C GenBank number: DQ345366.1
ATGGGCATGCGGATGATGTTCACCATGTTTCTGTTGGTTGTTTTGACAACCACTGTGGTTTCCTTCAATT
TAGATCGTGAATCCAATCACGAGAATCGCAGAACA-----------TCTAACCAGATTACGCGGGGCATGTGG
GATGAATGCTGTGACGATCCTCCATGTCGGCAAAATAATATGGAGCATTGTCCCGCAAGT------------------
----
>Qc1.11 GenBank number: JX293449
ATGGGCATGCGGATGATGTTCACCTTGTTTCTGTTGGCTGTCTTGTCAACCACTGTCGTTTCCTTCACTT
TAGATCGTGCATCTAATGGCAGGGATGCCGCAGCCGACAGCAAAGCGGCTGACCAGATCGCTCAGAC
CGTCAGGGATGAATGCTGTTCTAATCCTTCCTGTGCCCAGACTCATCCAGAGATTTGTCGCCGAACGCT
GATGCTCCAGAACCCTCTGAACCACGACATGTCGCCCTCTGCC
>Eb1.2 GenBank number: JX293431
ATGGGCATGCGGATGATGTTCACCTTGTTTCTGTTGGCTGTCTTGTCAACCACTGTCGTTTCCTTCACTT
TAGATCGTGCATCTAATGGCAGGGATGCCGCAGCCGACAGCAAAGCGGCTGACCAGATTGCTCAGACC
GTCAGGGATGAATGCTGTTCTAATCCTTCCTGTGCCCAGACTCATCCAGAGATTTGTCGCCGAACGCTG
ATGCTCCAGAACCCTCTGAACCACGACATGTCGCCCTCTGCC
>Rt1.2 GenBank number: JX293444
ATGGGCATGCGGATGATGTTCACCATGTTTCTGTTGGTTGTCTTGTCAACCACTGTCGTTTCCCTCCCTG
TAGATCATGCATCTAATGGCAGGGATGCCGCAGCCGACAGCAATGCGGCTGACCAGATCGCTCAGACG
GCCAGGGATCCATGCTGTTCTAATCCTTCCTGTGCCCAGACTCATCCAGAGATTTGTCGCCGA------ATG
CTCCAGAACCCTCTGAACCACGACATGTCACCCTCTGCC
>Lv1.10 GenBank number: JX293421
ATGGGCATGCGGATGATGTGCACCGTGCTTCTGTTGGTTGTCTTGTCAACCACTGTCGTTTCCCTCCCT
GTAGATCATGCATCTAATGGCAGGGATGCCGCAGCCGACAGCAATGCGGCTGACCAGATCGCTCAGAC
CGCCAGGGATCCATGCTGTTCTAATCCTTCCTGTGCCCAGACTCATCCAGAGATTTGTCGCCGA------AT
GCTCCAGAACCCTCTGAATCACGACATGTCGCCCTCTGCC

framework IV

>A4.1 GenBank number: FJ868062
--------------ATGTTCACCGTGTTTCTGTTGGTTGTCTTGGCAACCACTGTCGTTTCCATCCCTTCAGATCG
TGCATCTGATGGCAGGAATGCCGCAGTCAACGAGAGAGCGCCTTGGCTGGTCCCTTCGACAATCACG
ACTTGCTGTGGATATAATCCGGGGACAATGTGCCCTCCTTGCAGGTGCGATAATACCTGT------------------
----
>Sm4.2 GenBank number: FJ868096
--------------ATGTTCACCGTGTTTCTGTTGGTTGTCTTGGCAACCACTGTCGTTTCCATCCCTTCAGATCG
TGCATCTGATGGCAGGAATGCCGCAGTCAACGAGAGAGCGCCTTGGCTGGTCCCTTCGACAATCACG
ACTTGCTGTGGATATGATCCGGGGACAATGTGCCCTCCTTGCATGTGCAATAATACCTGTAAACCAACA
AAAAAA---AGACCAGGC------CGCAGAAACGAC
>SmIVB GenBank number: ACZ49783.1

---------------ATGTTCACCGTGTTTCTGTTGGTTGTCTTGGCAACCACTGTCGTTTCCATCCCTTCAGATCG
TGCATCTGATGGCAGGAATGCCGAAGTCAACGAGAGAGCGCCTTGGCTGGTCCCTTCGACAATCACG
ACTTGCTGTGGATATGATCCGGGGTCAATGTGCCCTCCTTGCATGTGCAATAATACCTGTAAACCAAAA
CCCAAA---AAATCAGGC------CGCAGAAACCAC

>A4.2 GenBank number: FJ868063

---------------ATGTTCACCGTGTTTCTGTTGGTTGTCTTGGCAACCACTGTCGTTTCCATCCCTTCAGATCG
TGCATCTGATGGCAGGAATGCCGCAGTCAACGAGAGACAATCTTGGCTGGTCCCTTCGACAATCACGA
CTTGCTGTGGATATGATCCGGGGACAATGTGCCCTCCTTGCAGGTGCAATAATACCTGTAAACCAAAAA
AACCA---AAACCAGGAAAAGGCCGCAGAAACGAC

>Cr4.1 GenBank number: FJ868074

---------------ATGTTCACCGTGTTTCTGTTGGTTGTCTTGGCAACCACTGTCGTTTCCATCCCTTCAGATCG
TGCATCTGATGGCAGGAATGCCGCAGTCAACGAGAGACAACCTTGGCTGGTCCCTTCGACAATCACG
ACTTGCTGTGGATATGATCCGGGGACAAAGTGCCCTCCTTGCAGGTGCAATAATACCTGTAAACCAAA
AAAACCA---AAACCAGGAAAAGGCCGCAGAAACGAC

>SmIVA GenBank number: ACZ49784.1

---------------ATGTTCACCGTGTTTCTGTTGGTTGTCTTGGCAACCACTGTCGTTTCCATCCCTTCAGATCG
TGCATCTGATGGCAGGAATGCCGCAGTCAACGAGAGACAAACTTGGCTGGTCCCTTCGACAATCACG
ACTTGCTGTGGATATGATCCGGGGACAATGTGCCCTACTTGCATGTGCGATAATACCTGTAAACCAAAA
CCCAAA---AAATCAGGC------CGCAGAAACGAC

>Sx4.1 GenBank number: FJ868108

---------------ATGTTCACCGTGTTTCTGTTGGTTGTCTTGGCAACCACTGTCGTTTCCATCCCTTCAGATCG
TGCATATGATGGCAAGAATGCCGCAGTCCACGAGAGACAATCTTGGCTGGTCCCTTCGACAATCACGA
CTTGCTGTGGTTATAGTCCGGGGACAATGTGCCCTCCTTGCATGTGCACTAATACCTGC---------------------
----

>Cn4.1 GenBank number: FJ868080

---------------ATGTTCACCGTGTTTCTGTTGGTTGTCTTGGCAACCACTGTCGTTTCCATCCCTTCAGATCG
TGCATCTGATGGCAGGAATGCCGCAGTCCATGAGAGAGCGCCTTGGCTGGTCCCTTCGCAAATCACGA
CTTGCTGTGGTTATAATCCGGGGACAATGTGCCCTTCTTGCATGTGCACTAATTCCTGC----------------------
----

>SIVA GenBank number: FJ868107.1

ATGGGCATGCGGATGATGTTCACCGTGTTTCTGTTGGTTGTCTTGGCAACCACTGTCGTTTCCACCCCT
TCAGATCGTGCATCTGATGGCAGGAATGCCGCAGTCCACGAGAGACAGAAGAGTCTGGTCCCTTCGG
TCATCACGACTTGCTGTGGATATGATCCGGGGACAATGTGCCCTCCTTGCAGGTGCACTAATAGCTGTG
GT--

>S4.4 GenBank number: JX293445.1

ATGGGCATGCGGATGATGTTCACCGTGTTTCTGTCGGTTGTCTTGGCAACCACTGTCGTTTCCACCCCT
TCGGATCGTGCATCTGATGGCAGGAATGCCGCAGTCCACGAGAGACAGAAGGGGCTGGTCCCTTCGG
TCATTACGACTTGCTGTGGATATGATCCGGGGACAATGTGCCCTCCTTGCAGGTGCACTAATTCCTGTC
CAAAAAAACCGAAA---AAACCAGGC------CGCAGAAACGAC

>SIVB GenBank number: FJ868106.1

---------------ATGTTCACCGTGTTTCTGTCGGTTGTCTTGGCAACCACTGTCGTTTCCACCCCTTCAGATCG
TGCATCTGATGGCAGGAATGCCGCAGTCCACGAGAGACAGAAGGAGCTGGTCCCTTCGGTCATCACG
ACTTGCTGTGGATATGATCCGGGGACAATGTGCCCTCCTTGCAGGTGCACTAATTCCTGTCCAACAAAA
CCGAAA---AAACCAGGC------CGCAGAAACGAC

>Ac4.1 GenBank number: FJ868059

--------------ATGTTCACCGTGTTTCTGTTGGTTGTCTTGGCAACCACTCTCGTTTCCATCCCTTCAGATCG
TGCATCTGATTTCAGGAATGCCGCAGTCCACGAGAGACAGAAGGAGCTGGTCGTTACGGCCACCACG
ACTTGCTGTGGTTATAATCCGATGACATCGTGCCCTCGTTGCATGTGCGATAGTAGCTGCAACAAG-------
--AAAAAACCAGGC------CGCAGAAACGAC

>Mn4.1 GenBank number: FJ868093

--------------ATGTTCACCGTGTTTCTGTTGGTTGTCTTGGCAACCACTCTCGTTTCCATCCCTTCAGATCG
TGCATCTGATTTCAGGAATGCCGCAGTCCACGAGAGACAGAAGGAGCTGGTCGTTACGGCCACCACG
ACTTGCTGTGGTTATAATCCGATGACATCGTGCCCTCGTTGCATGTGCGATAGTAGCTGCAACAAG-------
--AAAAAACCAGGC------CGCAGAAACGAC

>C4.2 GenBank number: FJ868067

ATGGGCATGCGGATGATGTTCACCGTGTTTCTGTTGGTTGTCTTGGCAACCACTGTCGTTTCCATCCCT
TCAGATCGTGCATCTGATGTCAGGAATGCCGCAGTCCACGAGAGACAGAAGGAGCTGGTCGTTACGG
CCACCACGACTTGCTGTGGTTATAATCCGATGTCAATGTGCCCTAAATGCATGTGCACTTATTCCTGTCC
CCACCAAAAGAAGAAAAGACCAGGC------CGCAGAAACGAC

>Cn4.2 GenBank number: FJ868081

--------------ATGTTCACCGTGTTTCTGTTGGTTGTCTTGGCAACCACTGTCGTTTCCATCCCTTCAGATCG
TGCATCTGATGTCAGGAATGCCGCAGTCCACGAGAGACAGAAGGATCTGGTCGTTACGGCCACCACG
ACTTGCTGTGGTTATAATCCGATGACAATATGCCCTCCTTGCATGTGCACTTATTCCTGTCCCCCC---AAA
AAGAAAAAAACCAGGC------CGCAGAAACGAC

>Cn4.3 GenBank number: FJ868082

--------------ATGTTCACCGTGTTTCTGTTGGTTGTCTTGGCAACCACTGTCGTTTCCATCCCTTCAGATCG
TGCATCTGAAGGCAGGAATGCCGTAGTCCACGAGAGAGCGCCTGAGCTGGTCGTTACGGCCACCACG
ACTTGCTGTGGTTATGATCCGATGACAATATGCCCTCCTTGCATGTGCACTCATTCCTGTCCACCA---AA
AAGAAAA---CCAGGC------CGCAGAAACGAC

>M4.2 GenBank number: FJ868086

--------------ATGTTCACCGTGTTTCTGTTGGTTGTCTTGGCAACCAGTGTCGTTTCCATCCCTTCAGATCG
TGCATCTGATGGCGGGAATGCCGTAGTCCACGAGAGAGCGCCTGAGCTGGTCGTTACGGCCACCACG
ACTTGCTGTGGTTATGATCCGATGACAATATGCCCTCCCTGCATGTGCACTCATTCCTGTCCACCA---AA
AGGAAAA---CCAGGC------CGCAGGAACGAC

>Cn4.4 GenBank number: FJ868083

--------------ATGTTCACCGTGTTTCTGTTGGTTGTCTTGGCAACCACTGTCGTTTCCATCCCTTCAGATCG
TGCATCTGATGGCAGGAATGCCGTAGTCCACGAGAGAGCGCCTGAGCTGGTCGTTACGGCCACCACG
ACTTGCTGTGGTTATGATCCGATGACATGGTGCCCTTCTTGCATGTGCACTTATTCCTGTCCCCAC---CA
AAGGAAAAAACCAGGC------CGCAGAAACGAC

>MIVA GenBank number: FJ868088.1

--------------ATGTTCACCGTGTTTCTGTTGGTTGTCTTGGCAACCACTGTCGTTTCCATCCCTTCAGATCG
TGCATCTGATGGCAGGAATGCCGTAGTCCACGAGAGAGCGCCTGAGCTGGTCGTTACGGCCACCACG
ACTTGCTGTGGTTTTGATCCGATGACATGGTGCCCTCCTTGCATGTGCACTTATTCCTGTTCCCAC---CA
AAGGAAAAAACCAGGC------CGCAGAAACGAC

>Ac4.2 GenBank number: FJ868060

--------------ATGTTCACCGTGTTTCTGTTGGTTGTCTTGGCAACCACTCTCGTTTCCATCCCTTCAGATCG
TGCATCTGATGGCAGGAATGCCGTAGTCCACGAGAGACAGCCTTGGCTGGTCCCTTCGAAAATCACGA
ATTGCTGTGGTTATAATAACATGGAAATGTGCCCTACTTGCATGTGCACTTATTCCTGTCGCCCC---AAA

AAGAAAAAACCAGGC------CACAGAAACGAC

>Mn4.2 GenBank number: FJ868094

--------------ATGTTCACCGTGTTTCCGTTGGTCGTCTTGGCAACCACTCTCGTTTCCATCCCTTCAGATCG
TGCATCTGATGGCAGGAATGCCGTAGTCCACGAGAGACAGCCTTGGCTGGTCCCTTCGAAAATCACGA
ATTGCTGTGGTTATAATACGATGGAAATGTGCCCTACTTGCATGTGCACTTATTCCTGTCGCCCC---AAA
AAGAAAAAACCAGGC------CGCAGAAACGAC

I1 superfamily

>Tx11.4 GenBank number: JX293451

ATGAAGCTGTGTGTGACGTTTCTTGTTGTTCTGGTGATTCTGCCATCAGTAACTGGGGAGAAGTCTAGC
GAGCGTACACTGAGTGGTGCTGCTCTGAGAGGCGATCGGAGAACGTGCTCAAACAAAGGACAACTAT
GCGGAGATGATTCCGACTGCTGTTGGCATTTGTGTTGTGTGGACAACAAGTGCGCTCACTTGATCCTAT
TATGTAACTTA---------

>S11.1 GenBank number: JX293453

ATGAAGCTGTGTGTGACGTTTCTTCTTGTTCTGGTGATTCTGCCATCAGTAACTGGGGAGGAGTCTAGC
GAGCGTACACTGAGTGGTGCTACTCTGACAGGCGGTCGGGGAATGTGCTCACTCTTAGGACAACGAT
GCGGAGATCATTCCGACTGCTGTTGGGACATGTGTTGTGCCAGCGAAATGTGCGTTGTGACTTTCCTTC
CATGTAAA------------

>Bt11.1 GenBank number: FJ959108.1

ATGAAGCTGTGTGTGGCGTTTCTTCTTGTTCTGGTGATTCTGCCATCGGTGATTGGGGGGAAGCCTAGC
GAGCGTACACTGAGTGGTGCTACTCGGAGAGGCGATCGGAGAATGTGCTTATCCCTAGGACAAAGATG
CGAACGTCATTCCAACTGCTGTGGCTATCTGTGTTGTTTCTACGACAAGTGTGTTGTGACTGCCATAGG
GTGTGGCCACTAC------

>Ep11.1 GenBank number: FJ959109.1

ATGAAGCTGTGTGTGACGTTTCTTCTTATTCTGGTGATTCTGCCATCGGTAACTGGGGAGAAGTCTAGC
AAGCGTACACTGAGTGGTGCTGCTCTGAGAGGCGATTGGGGAATGTGCTCAGGCATAGGACAAGGAT
GCGGACAAGATTCCAACTGCTGTGGGGATATGTGCTGTTATGGCCAAATATGCGCTATGACTTTCGCGG
CATGTGGTCCC---------

>Tx11.3 GenBank number: JX293452

ATGAAGCTGTGTGTGACGTTTCTTCTTGTTCTGGTGATTCTGCCATCAGTAACTGGGGTGAAGTCTAGC
GAGCGTACACTGAGTGGTGCTGCTCTGAGAGGCGATCGGGGAACGTGCTCAGGCAGAGGACAAGAAT
GCAAACATGATTCCGACTGCTGTGGGCATTTGTGTTGTGCCGGCATAACGTGCCAATTCACTTACATTC
CATGTAAA------------

>R11.3 GenBank number: FJ959113.1

ATGAAGCTGTGCCTGACGTTCCTTCTTGTTCTGATGATTCTGGCATCAGTGACTGGGGAGAAGTTAAGC
GAGCAAACACTGCGTCGTGCTGCTAGGAAAAACAAAGGCCCTCGATGCTGGGTCGGCCGTGTCCATT
GCACCTATCATAAAGACTGCTGTCCGTCGGTATGTTGTTTCAAGGGAAGGTGTAAACCACAATCATGG
GGATGCTGGTCGGGTCCGACC

O2 superfamily(framework VI/VII)

>lt7a GenBank number: DQ345373.1

ATGGAGAAACTGACAATCCTGCTTCTTGTTGCTGCTTTACTGATGTCGACCCAGGGCCTGATTCAAAGT

GGTGGA---GAAAACCGCCCAAAGGAGAAGATCAAATTTTTATCAAAGAGAAAAACAGTAGCTGAGAG
TTGGTGG---GAAGGCGAATGCTTGGGTTGGTCCAATTATTGTACTTCGCACAGCATTTGTTGTTCT---GG
TGAATGTATTCTT---AGT---TACTGCGATATTTGG--------------------

>Vn6.2 GenBank number: AF215005.1

ATGGAGAAACTGACAATCCTGCTTCTTGTTGCTGCTGTGCTGATGTCGACCCAGGCCCTGATTCAA-----
-------GAAAAACGCCCAAAGGAGAAGATCAAATTTTTATCAAAAAGAAAGTCAATTCCTGAGAGTTGGT
GG---GAGGGCGAATGCAGCGGTTGGTCCGTGCATTGTACTCAACACTCGGATTGTTGTTCT---GGTGAA
TGTACAGGT---AGT---TACTGCGAATTGTAT--------------------

>Vn6.1 GenBank number: AF215004.1

ATGGAGAAACTGACAATCCTGCTTCTTGTTGCTGCTGTGCTGATGTGGACCCAGGCCCTGATTCAA-----
-------GAAAAACGCCCAAAGGAGAAGATCAAATTTTTATCAAAAAGAAAGACAACTGCTGAGAGTTGG
TGG---GAGGGCGAATGCAGCGGTTGGTCCGTGTATTGTACTCAACACTCGGAGTGTTGTTCT---GGTGA
ATGTACAGGT---AAT---TACTGCGAATTGTTT--------------------

>Ts6.2 GenBank number: AF215013.1

ATGGAGAAACTGACAATCCTGCTCCTTCTTGCTGCTGTACTGGTGTTGGCCCAGGCCCTGATTAAAAA
AGGTGGTGGAGAAAAACGCCAAAAGGAGAAGATCAACTTTCTATCAAAAAGAAAGACAACTGCTGA
GAGTTGGTGG---GAGGGCGAATGCAGCGGTTGGTCCGTGTATTGTACGTCGGACCCGGAATGTTGTTCT
---GGTGAATGTAGCAGT---TAT---TACTGCGAATTGTGG--------------------

>Vn6.3 GenBank number: AF215007.1

ATGCAGAAACTGACAATCCTGCTTCTTGTTGCTGCTGTACTGATGTCGACCCAGGCCCTGATTAAAGGT
GGTGGA---GAAAAACGCCCAAAGGAGAAGATCAGATTTTTATCAAAAAGAAAGACAACTGCTGAGAG
GTGGTGG---GAAGGCGAATGCCGGGGTTGGTCCAATGGTTGTACGACGAACTCGGATTGTTGTTCT---A
ATAATTGTGATGGA---ACA---TTCTGCAAGTTGTGG--------------------

>Vn6.4 GenBank number: AF215008.1

ATGCAGAAACTGACAATCCTGCTTCTTGTTGCTGCTGTACTGATGTCGACCCAGGCCCTGATTAAAGGT
GGTGGA---GAAAAACGCCCAAAGGAGAAGATCAAATTTTTATCAAAAAGAAGGACAAATGCTGAGAG
GTGGTGG---GAGGGCGATTGCACAGGTTGGTTAGACGGTTGTACGTCGCCCGCGGAATGCTGTACT---G
CGGTTTGTGATGCG---ACC------TGCAAGTTGTGG--------------------

>Ts6.3 GenBank number: AF215015.1

ATGGAGAAACTGACAATCCTGCTCCTTGTTGCTGCTGTACTGATGTCGACCCAGGCCCTGATTCAAAG
AGGTGGA---GCAAAACGC------CGAAAGGTCAACTTTTTTTCAATAAGAGAGCCAGGTGCTGAGGATTG
GAGG---GAGGGCAATTGCACACCCTGGTTAGGGGGATGTACGTCACCCGAGGAATGCTGTCCT---GGG
AATTGTGAGACG---------TACTGCAGGGCGTGGCGA-----------------

>LeD51 GenBank number: DQ141156.1

ATGGAGAAACTGACAATCCTGCTTCTTGTTGCTGCTGTACTGATGTCGACCCAGGCCCTGGTTGAACG
TGCTGGA---GAAAACCGCTCAAAGGAGAACATCAAATTTTTATTAAAAAGAAAAAGAGCTGCTGACA
GAGGGATG---TGGGGCAAATGCAAAGATGGGTTAACGACATGTCTTGCGCCCTCGGAGTGTTGTTCC---
GGGAATTGTGAACAG---------AACTGCAAGATGTGG--------------------

>TxMEKL-011 GenBank number: AF215017.1

ATGGAGAAACTGACAATCCTGCTTCTTGTTGCTGCTGTACTGATGTCGACCCAGGCCCTGGTTGAACG
TGCTGGA---GAAAACCGCTCAAAGGAGAACATCAAATTTTTATTAAAAAGAAAAAGAGCTGCTGACA
GAGGGATG---TGGGGCAAATGCAAAGATGGGTTAACAACATGTCTTGCGCCCTCGGAGTGTTGTTCC---
GGGAATTGTGAACAG---------AACTGCAAGATGTGG--------------------

>Pn6.9 GenBank number: AF215031.1

ATGGAGAAACTGACAATCCTGCTTCTTGTTGCTGCTGTACTGATGTCGACCCAGGCCCTGCCTCAGGG
TGGTGGA---GAAAACCGCCTAAAGGAGAACATCAAATTTTTATTAAAAAGAAAGACAGCTGCTGACAG
GGGGATG---TGGGGCGATTGCGATGATTGGTTAGCAGCATGTACTACGCCCTCGCAGTGTTGTACT---GA
AGTTTGTGATGGG---------TTCTGCCGCCTATGGGAA------------------

>TxMEKL-04111 GenBank number: AF215021.1
ATGCAGAAACTGATAATCCTGCTTCTTGTTGCTGCTGTGCTGATGTCGGCCCAGGCCGTGCTTCAA------
------GAAAAACGCCCAAAGGAGAAGATCAAGTTTTTATCAAAGAGAAAGACAGATGCTGAGAAGCAG
CAG---AAGCGCCTTTGCCCGGATTACACGGAGCCTTGTTCACATGCCCATGAATGCTGTTCA---TGGAAT
TGTTATAATGGGCAC------TGTACGGGA------------------------

>TxMEKL-0421 GenBank number: AF215023.1
ATGCAGAAACTGATAATCCTGCTTCTTGTTGCTGCTGTGCTGATGTCGACCCAGGCCGTGCTTCAA------
------GAAAAACGCCCAAAGGAGAAGATCAAGTTTTTATCAAAGAGAAAGACAGATGCTGAGAAGCAG
CAG---AAGCGCCTTTGCCCGGATTACACGGATCCTTGTTCACATGCCCATGAATGCTGTTCA---TGGAAT
TGTTATAATGGGCAC------TGCACGGGA------------------------

>Pn6.8 GenBank number: AF215030.1
ATGCAGAAACTGATAATCCTGCTTCTTGTTGCTGCTGTGCTGATGTCGACCCAGGCCCTGTTTCAA------
------GAAAAACGCCTAAAGGAGAAGATCAATTTTTTATCAAAGGAAAAGGCAGATGCTGAGAAGCAGC
AG---AAGCGCTATTGCTCGGATCAATGGAAGTCTTGTTCATATCCCCACGAATGCTGTAGA---TGGAGTT
GT---AATAGGTAC------TGCGCG---------------------------

>TxMEKL-043 GenBank number: AF215024.1
ATGCAGAAACTCATAATCCTGCTTCTTGTTGCTGCTGTGCTGATGTCGACCCAGGCCCTGTTTCAA-------
-----GAAAAACGCCCAATGAAGAAGATCGATTTTTTATCAAAGGGAAAGACAGATGCTGAGAAGCAGC
AG---AAGCGCAGTTGCTCGGATGATTGGCAGTATTGTGAAAGTCCCACTGACTGCTGTAGT---TGGGAT
TGT---GATGTGGTC------TGCTCGGGA------------------------

>TxVIIA GenBank number: AF215019.1
ATGGAGAAACTGACAATCCTGCTCCTTGTTGCTGCTGTACTGATGTCGACCCAGGCCATGTTTCAAGGT
GATGGA---GAAAAATCCCGGAAGGCGGAGATCAACTTTTCTGAAACAAGAAAGTTGGCGAGAAACAA
GCAGAAA---------CGCTGCGGTGGTTATTCAACGTATTGTGAAGTTGACTCGGAATGCTGTTCC---GACA
ATTGTGTAAGGTCTTAC------TGCACGCTGTTTGGA------------------

>MaI51 GenBank number: DQ141159.2
ATGCAGAAACTGACAATCCTGCTTCTTGTTGCTGCTGTGCTGCTGTCGACCCAGGCCCTAAATCAA-----
-------GAAAAACGCCCAAAGGAGATGATCAATGTTTTATCAAAAGGAAAGACAAATGCTGAGAGGCGG-
-----AAGCGCCAATGCGAGGATGTTTGGATGCCTTGTACATCGAACTGGGAATGCTGTTCT---TTGGATTG
T---GAAATGTAC------TGCACACAGATAGGA------------------

>lt7b GenBank number: DQ512967.1
ATGGAGAAACTGACAATCCTGCTTCTTGTTGCTGCTTTACTGATGTCGACCCAGGGCCTGATTCAAGA
AAAACGC---CAAAAG---------GCGAAGATCACCATTTTTTCAAAAAGGAAGTCAAATGCTGAGAGGTGG
TGG---GAGGGCGATTGCACTGATTGGTTAGGGTCGTGTTCATCGCCCTCGGAGTGTTGTTAT---GACAAT
TGTGAAACG---------TACTGCACGTTGTGGAAA------------------

>TxMEKL-021 GenBank number: AF215027.1
ATGGAGAAACTGACAATCCTGCTTCTTGTTGCTGTTGTACTGATGTCGACCCAGGCCCTGCCTCAAGG
TGGTGGA---GAAAAACGCCCAAGGGAGAATATCAGATTTTTATCAAAAAGAAAGTCAAATGCTGAGCG
TTGGAGG---GAGGGCAGTTGCACCTCTTGGTTAGCGACGTGTACGGACGCCTCGCAATGCTGTACT---G
GTGTTTGTTACAAAAGGGCC---TACTGCGCCTTGTGGGAA------------------

>Tx7.30 GenBank number: JX293455
ATGGAGAAACTGACAATTCTGCTTCTCGCTGCTACTGTGCTGATGTCGACCCAGGCCCTAATTCAA------
------GAACAAAGCCAAAAGGCAGAGATCAACTTGTTTTCAAAGAGGAAGCCATCTGCTGAACGTTGGC
GGGTAGACAGCGAATGCATTTCTTTTTGGGGGTCTTGTACGGTGGACGCGGATTGTTGTTTT---AATAGT
TGTGACGAAACGTATGGCTACTGC-----------------------------
>Tx7.31 GenBank number: JX293558
ATGGAGAAACTGACAATTCTGCTTCCCGTTGCTGCTGTGCTGATGTCGACCCAGGCCCTAATTCAA------
------GAACAACGCCAAAAGGCAAAGATCAACTTGTTTTCAAAGAGAAAGCCATCTGCTGAGCGTTGGT
GGGGAGAGAATGACTGC---AGTTGGACAGGGCCTTGTACGGTGAACGCAGAATGTTGTCTT---GGTGT
TTGTGATGAAACG---------TGC-----------------------------
>Tx7.29 GenBank number: JX293557
ATGGAGAAACTGACAATTCTGCTTCTTGTTGCTGCTGTGCTGATGTCGACCCAGGCCCTAATTCAA------
------GATCAACGCCAAAAGGCAAAGATCAACTTGTTTTCAAAGAGACAGGCATATGCTCGTGATTGGTG
G---GACGATGGCTGC---AGTGTGTGGGGGCCTTGTACGGTGAACGCAGAATGTTGTTCT---GGTGATTGT
CATGAAACG---------TGC-----------------------------
>Mi7.6 GenBank number: JX293456
ATGGAGAAACTGACAATCCTGCTTCTTGTTGCTGCTGTACTGTTGTCGATCCAGGCCCTAAATCAA------
------GAAAAACACCAACGGGCAAAGATCAACTTGCTTTCAAAGAGAAAGCCACCTGCTGAGCGTTGGT
GGCGGTGGGGAGGATGCATGGCTTGGTTTGGGCTTTGTTCGAGGGACTCGGAATGTTGTTCT---AATAG
TTGTGACGTAACG---------TGC-----------------------------
>TxMEKL-0511 GenBank number: AF215025.1
ATGGAGAAACTGACAATTCTGCTTCTTGTTGCTGCTGTACTGTTGTCGATCCAGGCCCTAAATCAA------
------GAAAAACACCAACGGGCAAAGATCAACTTGCTTTCAAAGAGAAAGCCACCTGCTGAGCGTTGGT
GGCGGTGGGGAGGATGCATGGCTTGGTTTGGGCTTTGTTCGAAGGACTCGGAATGTTGTTCT---AATAG
TTGTGACGTAACGCGC------TGCGAGTTAATGCCATTCCCACCAGACTGG---
>PnVIIA GenBank number: AF215029.1
ATGGAGAAACTGACAATTCTGCTTCTTGTTGCTGCTGTACTGATGTCGACCCAGGCCCAAAATCAA-----
-------GAACAACGCCAACAGGCAAAGATCAACTTCCTTTCAAAGAGAAAGCCATCTGCTGAGCGTTGG
AGGAGA------GATTGCACTTCTTGGTTTGGGCGTTGTACAGTGAACTCGGAATGTTGTTCT---AATAGTT
GTGACCAAACGTAC------TGCGAGTTATATGCATTCCCATCATTCGGCGCC
>Tr7.2 GenBank number: JX293458
ATGGAGAAACTGACAATCCTGCTTCTCCATGCTGCTGTACTGATGTCGACCCAGGCCCTGATTCAAGGT
GGTGGA---GAAAAACGCCCAAAGGAGAAGATCAATTTTTTATCAAAAAGAAAGATAGCTGCTAAGAGA
TGGTGG---CTCGGGGAATGCGATGAATGGGACTGGCTGTGTGATCAACCTTCAGAATGTTGCTCT---GC
TACTTGTTCCTTGTGG---------TGC-----------------------------
>Tr7.1 GenBank number: JX293457
ATGGAGAAACTGACAATTCTGCTTCTTGTTGCTGCTGTACTGACGTCGACCCAGGCCCTGATTCAAGAT
CGT------AAAGAACGTCAAAAGGCGGAGATCAACTTTTTGTCAAAAAGAAAATCAACTTTTTGGCGTT
GGTGG---GATGGCGATTGCAGGACTTGGCGTGCGCCGTGTAACCCTGGCGTGGAATGTTGT---ACTGAT
GTATGT------AGACACGGCCGCTGTGTGTTCTGG--------------------
>Vn6.5 GenBank number: AF215009.1
ATGCAGAAACTGACAATCCTGCTTCTTGTTGCTGCTGTACTGATGTCGACCCAGGCCCTGATTCGAGGT
GGTGTA---GAAAAACGTCAAGAGGCGAAGAGGAACTTTTTTTCAAAAAGAAAGACAACTGCTGAGAG
TTGGTGG---GAGGGCGAATGCAGGACTTGGTATGCGCCGTGTAATTTTCCCTCGCAATGCTGTTCT---GA

GGTTTGTAGCAGCAAAACTGGCCGCTGCCTGACGTGG---------------------

O3 superfamily

>ArMSGL-0143 GenBank number: AF215073.1
ATGTCAGGATTGGGAATCATGTTGCTAACCCTTCTACTTCTTGTGTTCATGGAAACCAGTCATCAGGAT
GCAGGAGAGAAGCAGGCGACGCAAAGGGACGCAATCAACGTCAGACGGAGAAGATCACTCACTCGG
AGA------GTAACTGAGGAGTGCGAAGAGAACTGTGAGGAGGAGGAAAAG---CACTGCTGCAACACAA
ATAATGGACCC---TCTTGTGCCCCGCAATGCTTCGGA

>Tx7.32 GenBank number: JX293468
ATGTCAGGATTGGGAATCATGGTGCTCACCCTTCTACTTCTTGTGTTCATGGCAACCAGTCATCAGGAT
GCAGGAGAGAAGCAGGCCACGCAAAGGGACGCAATCAACGTCAGACGGAGAAGATCACTCGCTCGG
AGAACA---GTAACTGAGGAGTGCGAAGAGGACTGTGAGGATGAGGAAAAG---CACTGCTGCAACACA
AATAATGGACCC---TCTTGTGCCCGTCTATGCTTCGGA

>ArMSGL-0141 GenBank number: AF215070.1
ATGTCAGGATTGGGAATCTTGGTGCTAACCCTTCTACTTCTTGTGTACATGGCAACCAGTCATCAGGAT
GCAGGAGAGAAGCAGGCGACGCAAAGGGACGCAATCAACGTCAGACGGAGAAGATCACTCACTCGG
AGA------GTAGCTGAGGAGTGCGAAGAGTCCTGTGAGGATGAGGAAAAG---CACTGCTGCAACACAAA
TAATGGACCC---TCTTGTGCCCCGCAATGCTTCGGA

>PnMSGL-01 GenBank number: AF215074.1
ATGTCAGGATTGGGAATCATGGTGCTAACCCTTCTACTTCTTGTGTTCATGGAAACCAGTCATCAGGAT
GCAGGAGAGAAGCAGGCGATGCAAAGGGACGCAATCAACGTCAGACGGAGAAGATCAATCACTCGG
AGA------GTATCTGAGGCGTGCGAAGAGTCCTGTGAGGATGAGGAAAAA---CACTGCTGCCACGAAAA
TAATGGAGTATACACTTGTCTCCGCTATTGCTGGGGA

>ArMSGL-011 GenBank number: AF215072.1
ATGTCAGGATTGGGAATCATGGTGCTAACCCTTCTACTTCTTGTGTTCATGGAAACCAGTCATCAGGAT
GCAGGAGAGAAGCAGGCGATGCAAAGGGACGCAATCAACGTCAGACGGAGAAGATCAATCACTCGG
AGA------GTATCTGAGGCGTGCGAAGAGTCCTGTGAGGAGGAGGAAAAA---CACTGCTGCCACGAAAA
TAATGGAGTATACACTTGTCTCCGTTATTGCTGGGGA

>S6.18 GenBank number: JX293474
ATGTCAGGATTGGGAATCATGGTGCTCACCCTTCTACTTCTTGTGATCATGGCAACCAGTCATCAGGAT
GCAGGAGAGAAGCAGATGACGCAAAGGGACGCAATCAACGTCAGACGGAGAAGATCAATCACTGGG
AGAGTA---GTAACTGAGGCGTGCGAAGAGGCCTGTGAGCAGGAGGAGAAG---AACTGCTGTGGCATA
ACAAATGGACAACCCTTTTGTAACTTTCCATGCATCGGA

>S6.17 GenBank number: JX293472
ATGTCAGGATTGGGAATCATGGTGCTAACCCTTCTACTTCTTGTGTTCATGGCAACCAGTCGTCAGGAT
GCAGGAGAGAAGCAGGCGACGCAAAGGGACGCAATCAAAGTCATA---CGGAGATCAGTCATTCAGAG
AAAA---GCAACTGAGGAGTGCGAAGAGGTCTGTGAGCAGGATGAAAAG---CACTGCTGTGACGGAAG
TGATGGAACCCCCAGATGTACCAGTCGTTGCTTTGGA

>TsMSGL-12 GenBank number: AF215064.1
ATGTCAGGATTGGGGATCATGGTGCTAACCCTTCTACTTTTTATGTTCATGGCAACCAGTCATCAGGATG
CAGGAGAGAAGCAGGCGACGCAAAGGGACGCAATCAACGTCAGACGGAGAAGATCAATCACTCGGA
GA------GGAGATGAGGAGTGCAATGAGTACTGTGACGATCGGAACAAG---GAGTGCTGCGGCAGAACA
AATGGACATCCCCGATGTGCCAATGTATGCTTTGGA

>TsMSGL-13 GenBank number: AF215063.1
ATGTCAGGATTGGGGATCATGGTGCTAACCCTTCTACTTTTTATGTTCATGGCAACCAGTCATCAGGATG
CAGGAGAGAAGCAGGCGACGCAAAGGGACGCAATCAACGTCAGACGGAGAAGATCAATCACTCGGA
GA------GGAGATGAGGAGTGCAATGAGCACTGTGAGGATCGGAACAAG---GAGTGCTGCGGCAGAACA
AATGGACATCCCCGATGTGCCAATGTATGCTTTGGA
>TsMSGL-11 GenBank number: AF215062.1
ATGTCAGGATTGGGAATCATGGTGCTAACCCTTCTACTTCTTGTGTTCATGGCAACCAGTCATCAGGAT
GCAGGAGAGAAGCAGGCGACGCAAAGGGACGCAATCAACGTCAGACGGAGAAGATCAATCACTCGG
AGA------GTAGATGAGGAGTGCAATGAGTACTGTGACGATCGGAACAAG---GAGTGCTGCGGCAGAAC
AAATGGACATCCCCGATGTGCCAATGTATGCTTTGGA
>S6.16 GenBank number: JX293473
ATGTCAGGATTGGGAATCATGGTGCTAACCCTTCTACTTCTTGTGTTCATGGCAACCAGTCATCAGGAT
GCAGGAGAGAAGCAGGCGACGCAAAGGGATGCAATCAGCGTCATAGGGAGAAGATCAATCATTCGGA
GACGA---GTAGATGAGGAGTGCAATGAGATCTGTGGGGAACAGGGGAAG---AACTGCTGCGGCAGAA
GTAATGGAACCCCCAGATGTGCCAAGGTTTGCTTTGGA
>VnMSGL-0111 GenBank number: AF215080.1
ATGTCAGGATTGGAAATTATGGTGCTAACCCTTCTACTTCTTGTGTCCATGGCAACCAGTCATCAGGAT
GGAGGAGAGAAGCAGGCGACACAAAGGGACGCAATCAACGTCAGACGGAGA---TCAATCACTCGA---
---------ACTGAGGCGTGCTATGAGTACTGTAAGGAGCAGAACAAG---ACCTGCTGTGGCATAAGCAATG
GACGTCCCATTTGTGTCGGTGGATGCATCAGA
>VnMSGL-0112 GenBank number: AF215078.1
ATGTCAGGATTGGGAATCATGGTGTTAACCCTTCTACTTCTTGTGTCCATGGCAACCAGTCATCAGGAT
GGAAGAGGGAAGCAGGCGACGCAAAGGGACGCAATCAACGTCAGACGGAGAAGATCAATCACTCGA
------------ACTGAGGCGTGCTATGAGTACTGTAAGGAGCAGAACAAG---ACCTGCTGTGGCATAAGCAAT
GGACGTCCCATTTGTGTCGGTGGATGCATCAGA
>VnMSGL-0123 GenBank number: AF215079.1
ATGTCAGGATTGGGAATCATGGTGTTAACCCTTCTACTTCTTGTGTCCATGGCAACCAGTCATCAGGAT
GGAGGAGGTAAGCAGGCGACGCAAAGGGACGCAATCAACGTCAGACGGAGAAGATCAATCACTCGG
AGAGAAGTAGTAACTGAGGAGTGCGAAGAGTACTGTAAGGAGCAGAACAAG---ACCTGCTGCGGCCT
AACAAATGGAAGACCCAGATGTGTCGGGGTTTGCTTCGGA
>VnMSGL-0121 GenBank number: AF215076.1
ATGTCAGGATTGGGAATCATGGTGTTAACCCTTCTACTTCTTGTGTCCATGGCAACCAGTCATCAGGAT
GGAGGAGGGAAGCAGGCGACGCAAAGGGACGCAATCAACGTCAGACGGAGAAGATCAATCACTCGG
AGA---GTAGTAACTGAGGCGTGCGAAGAGTACTGTGAGGACCGGGACAAGAAGACATGCTGCGGCCT
AGAAAATGGAGAACCCTTTTGTGCCACTCTATGCTTCGGA
>VnMSGL-0122 GenBank number: AF215077.1
ATGTCAGGATTGGGAATCATGGTGTTAGCCCTTCTACTTCTTGTGTTCATGGCAACCAGTCATCAGGAT
GGAGGAGGGAAGCAGGCGACGCAAAGGGACGCAATCAACGTCAGACGGAGAAGATCAATCACCCGG
AGA---GTAGTAACTGAGACGTGCAAAGAGTACTGTGAGGACCGGGACAAG---ACCTGCTGCGGCCTAG
AAAATGGACAACCCGATTGTGCCAATCTATGCCTCGGA
>ArMSGL-0122 GenBank number: AF215071.1
ATGTCAAGATTGGGAATCATGGTGCTAACCCTTCTACTTCTTGTGTTCATAGTAACCAGTCATCAGGATG
CAGGAGAGAAGCAGGCGACGCAAAGGGCCGCAATCAACTTCAGATGGAAAAGATCACTCACTCGGA
GA---ACAGCAACTGAGGAGTGCGAAGAGTACTGCGAAGATGAGGAAAAG---ACCTGCTGCGGCGAAG

AAGATGGAGAACCCGTATGTGCCCGCTTTTGCTTAGGA

>ArMSGL-0123 GenBank number: AF215068.1

ATGTCAAGATTGGGAATCATGGTGCTAACTCTTCTACTTCTTGTGTTCATAGTAACCAGTCATCAGGATG
CAGGAGAGAAGCAGGCGACGAAAAGGGCCGCAGTCAACTTCAGATGGAGAAGATCATTCACTCGGA
GA---GCAGCAGCTGAGGAGTGCGAAGAGTACTGTGAGGAGGAGGAAAAG---ACCTGCTGCGGCGAAG
AAGATGGAGAACCCGTATGTGCCGAATTTTGCTTAGGA

>ArMSGL-0124 GenBank number: AF215066.1

ATGTCAAGATTGGGAATCATGGTGCTAACCCTTCTACTTCTTGTGTACATGGCAACCAGTCATCAGGAT
GCAGGAGAGAAGCAGGCGACGCAAAGGGACGCAATCAACTTCAGATGGAAAAGATCACTCACTCGG
AGA---ACAGCAACTGAGGAGTGCGAGGAGTCCTGTGAGGAGGAGGAAAAG---ACCTGCTGCGGCGAA
?AAGATGGAGAACCCGTATGTGCCCGCTTTTGCTTAGGA

>PnMSGL-03 GenBank number: AF215075.1

ATGTCAAGATTGGGAATCATGGTGCTAACCCTTCTACTTCTTGTGTTCATAGTAACCAGTCATCAGGATG
CAGGAGAGAAGCAGGCGACGCAAAGGGACGCAATCAACTTCAGATGGAGAAGATCACTCATTCGGA
GA---ACAGCAACTGAGGAGTGCGAAGAGTACTGTGAGGATGAGGAAAAG---ACCTGCTGCGGCTTAG
AAGATGGAGAACCCGTATGTGCCACGACATGCCTCGGA

>ArMSGL-021 GenBank number: AF215069.1

ATGTCAAGATTGGGAATCATGGTGCTAACTCTTCTACTTCTTGTGTTCATAGTAACCAGTCATCAGGATG
CAGGAGAGAAGCAGGCGACGCATAGGGGCGCAATCAACTTCAGATGGAGAAGATCACTCATTCGGAG
A---ACAGCAACTGAGGAGTGCGAAGAGTACTGTGAGGATGAGGAAAAG---ACCTGCTGCGGCTTAGA
AGATGGAGAACCCGTATGTGCCACGACATGCCTCGGA

>ArMSGL-0121 GenBank number: AF215067.1

ATGTCAAGATTGGGAATCATGGTGCTAACCCTTCTACTTCTTGTGTTCATAGTAACCAGTCATCAGGATG
CAGGAGAGAAGCAGGCGACGCAAAGGAACGCAATCAACTTCAGATGGAGAAGATCATTCACTCGGA
GA---GCAGCAACTGAGGAGTGCGAAGAGTACTGCGAAGATGAGGAAAAG---ACCTGCTGCGGCTTAG
AAGATGGAGAACCCGTATGTGCCACGACATGCCTCGGA

>Tr7.4 GenBank number: JX293470

ATGTCAAGATTGGGAATCATGGTGCTAACCCTTCTACTTCTCGTGTTCATGGTAACCAGTCATCAGGAT
AGTAGAGAGAAGCAGGCGACTCGAAGGAACGCAGTCAACATCGGATGGAGAAGATCAATTATTCGGA
GA---ACAGTAGGTGAGGAGTGCAACGAGTACTGTGAGCAGCGGAACAAG---AACTGCTGCGGTAAAA
CAAATGGAGAACCCGTATGTGCACAGGCATGCCTCGGA

>Tr7.5 GenBank number: JX293471

ATGTCAGGATTGGGAATCATGGTGCTAACCCTTCTACTTCTCGTGTTCATGGTAACCAGTCATCAGGAT
AGTGGAGAGAAGCGGGCGACTCGAAGGAACGCAATCAACATCGGATGGAGAAGATCAATCATTCGGA
GAGCATCATTAGATGAGGAGTGCAACGAGTACTGCAAGCAGCGGACCAAG---AACTGCTGCGGTAAA
ACAAATGAAGAACCTGTATGTGCACGGCGCTGCCTCGGA

>Tr7.3 GenBank number: JX293469

ATGTCAGGATTGGGAATCATGGTGCTAACCCTTCTACTTCTCGTGTTCATGGTAACCAGTCATCAGGAT
AGTGGAGAGAAGCAGGCGACTCGAAGGAACGCAGTCAACATCGGATGGAGAAGATCAATTATTCGGA
GA------ACAACAGAAGAGTGCCATGAGTACTGTGAGGATCAGAACAAG---AACTGCTGCGGATTAACA
GATGGAGAACCCAGATGTGCTGGGATGTGCCTTGGA

>TsMSGL-2 GenBank number: AF215065.1

ATGTCAGGATTGGGAATCATGGTGCTAACCCTTCTACTTCTTGTGTTCATGGCAACCAGTCATCAGGAT
GCAGGAGAGAAGCAGGCGACGCAAAGGGACGCAGTCAACGTCAGACGGAGAAGATCAATCGCTGGG

AGAACA---ACAACCGAGGAGTGCGATGAGTACTGTGAGGATCTAAACAAG---AACTGCTGCGGCTTAT
CAAATGGAGAACCCGTATGTGCCACGGCATGCCTCGGA

S superfamily

>S8.1 GenBank number: JX293475
ATGATGTTGAAAATGGGAGCTATGTTTGTCCTTTTGCTTCTTTTCATCCTGCCATCCAGCCAGCAGGAA
GGAGATGTCCAGGCAAGAAAAACGCACCTGAAGAGAGGCTTCTACGGTACTCTGGCAATGTCTACCA
GAGGATGCTCT---GGCACTTGCCATCGTCGTGAGGACGGCAAGTGTCGGGGTACTTGCGACTGCTCCG
GATACAGCTATTGTCGCTGCGGT---GACGCTCACCATTTTTACCGAGGATGCACGTGTTCATGTCAAGGT

>Ca8c GenBank number: EU516351.1
ATGATGTTGAAAATGGGAGCTATGTTTGTCCTTTTGCTTCTTTTCATCCTGCCATCCAGCCAGCAGGAA
GGAGATGTCCAGGCAAGAAAAACGCACCTGAAGAGCGGCTTCTACGGTACTCTGGCAATGTCTACCA
GAGGATGCTCT---GGCACTTGCCGTCGTCATCGGGACGGCAAGTGTCGGGGTACTTGCGAGTGCTCCG
GATACAGCTATTGTCGCTGCGGT---GACGCTCACCATTTTTACCGAGGATGCACGTGTACATGT---------

>Ca8a GenBank number: EF208032.2
ATGATGTTGAAAATGGGAGCTATGTTTGTCCTTTTGCTTCTTTTCATCCTGCCATCCAGCCAGCAGGAA
GGAGATGTCCAGGCAAGAAAAACGCACCTGAAGAGAGGCTTCTACGGTACTCTGGCAATGTCTACCA
GAGGATGCTCT---GGCACTTGCCATCGTCGTGAGGACGGCAAGTGTCGGGGTACTTGCGACTGCTCCG
GATACAGCTATTGTCGCTGCGGT---GACGCACACCATTTCTATCGAGGATGCACGTGTACATGT---------

>Tx8.1 GenBank number: JX293526
ATGATGTTGAAAATGGGAGCTATGTTTGTCCTTTTGCTTCTTTTCACCCTGGCATCCAGCCAGCAGGAA
GGAGATATCCAGGCAAGGAAAACACACCTGAAGAGCGGCTTCTATCGTACTCTGCCAAGGTTTGCCA
GAGGATGCACT---ATCTCTTGTGGA---TATGAAGACAACAGGTGTCAGGGTGAATGCCACTGCCCCGGA
AAAACCAATTGTTACTGTACC---AGTGGCCATCATAAC---AAAGGATGCGGTTGTGCATGT---------

>S8.2 GenBank number: JX293476
ATGATGTTGAAAATGGGAGCTATGTTTGTCCTTTTGCTTCTTTTCACCCTGGCATCCGTCCAGCAGGAA-
--GATGTCCAGGCAAGGAAAACACGCCTGAAGAGAGACTTCCATCGTGCTCTGAGACAGTCTCCCTTA
GGATGCCCT---GGCACTTGTGGT------AAAGGCGGCAAGTGTGTGGGGTCTTGCGAATGCACTATGTATA
AAAATTGTTACTGCAGT---AACAATGGCATACGTCAACCAGGATGCTCGTGTACATGTCCAACTCGT

>RVIIIA GenBank number: FJ959114.1
ATGATGTCGAAAATGGGAGCTATGTTTGTCCTTTTGCTTCTTTTCACCCTGGCATCCAGCCAGCAGGAA
GGAGATGTCCAGGCAAGGAAAACACACCCGAAGAGAGAGTTCCAACGTATTCTGCTAAGGTCTGGCA
GAAAGTGCAATTTCGACAAATGTAAAGGTACCGGAGTCTACAATTGTGGGGAATCCTGCTCATGCGAA
GGTTTGCACAGTTGTCGCTGCACTTATAACATCGGTTCTATGAAGTCTGGATGCGCGTGTATTTGTACAT
ACTAT

T superfamily

>S5.3 GenBank number: JX293484
ATGCGCTGTTTCCCAGTCTTCGTTATTCTTCTGCTGCTGATTGCATCTGCACCTAGTGTTGATGCCCGAC
CGAAGACCAAAGATGGTATGCCCCTGGCATCTTTCCACGATAATGCAACTCGAAACCTGCAAATACTT--
-------------TGCTGCAAACATACCCCCGCATGCTGTACAGGAAAA---------

>Eb5.5 GenBank number: JX293523
ATGCGCTGTTTCCCAGTCTTCGTTATTCTTCTGCTGCTGATTGCATCTGCACCTAGTGTTGATGCCCGAC
CGAAGACCAAAGATGGTATGCCCCTGGCATCTTTCCACGATAATGCAACTCGAAACCTGCAAATACTT--
-------------TGCTGCAAGCATACCCCCGCATGCTGTACAGGAAAA---------
>Eb5.4 GenBank number: JX293522
ATGCGCTGTCTCCCAGTCTTCGTTATTCTTCTGCTGCTGATTGCATCTGCACCTAGTGTTGATGCCCGAC
CGAAGACCAAAGATGGTATGCCCCTGGCATCTTTCCACGATAATGCAACTCGAAACCTGCAAATACTT--
-------------TGCTGCAAACATACCCCCAAATGCTGTACAGGAAAA---------
>Eb5.2 GenBank number: JX293520
ATGCGCTGTTTCCCAGTCTTCGTTACTCTTCTGCTGCTGATTGCATCTGCACCTAGTGTTGATGCCCGAC
CGAAGACCAAAGATGGTATGCCCCTGGCATCTTTCCACGATAATGCAACTCGAAACCTGCAAATACTT--
-------------TGCTGCAAACATTCCCTCGAATGCTGTACAGGAAAA---------
>Eb5.3 GenBank number: JX293521
ATGCGCTGCTTCCCAGTCTTCGTTATTCTTCTGCTGCTGATTGCATCTGCACCTAGTGTTGATGCCCGAC
CGAAGACCAAAGATGGTATGCCCCTGGCATCTTTCCACGATAATGCAACTCGAAACCTGCAAATACTT--
-------------TGCTGCAAACATACCCCCGCATGCTGTAAA---------------
>Mi5.2 GenBank number: JX293487
ATGCGCTGCCTCCCAGTCTTCATCATTCTTCTGTTGCTGATTCCATCTGCATCTAGCGTTGATGTCCAAC
CGTTGACCAAAGATGATGTGCCCCTGGCATCTTTCCTCGATGATGCAAGGCGAACCCTACAAAGTCTC
TGGATGACACGCAGATGCTGTCCAGGAAATTTTGCGTGCTGTGGAAAA------------
>Cap5.1 GenBank number: JX293500
ATGCGCTGCTTCCCAGTCTTCATCATTCTTCTGTTACTGATTTCATCTGCATCTAACGTTGATGCCCAAC
AGAAGACCAAAGATGATGCGTCCCTGGCATCTTTCCAAGATAATGCAAGGCGAACCCTACAAAGTCTC
TGGATGACACGCGGATGCTGTCCAGGAAATGTTTTGTGCTGTGGAAAA------------
>Ca5.3 GenBank number: EU090174.1
ATGCGCTGTGTCCCAGTCTTCATCATTCTTCTGCTGCTGATTGCATCTGCACCTGGCGTTGATGCCCAAC
CGAAGACCAAATATAATGCGCCCCTGACATCTCTCCACGATAATGCAAAGGGTATACTACAAGAACATT
GGAACAAACGC---TGCTGCCCCAGAAGGCTTGCCTGCTGTATAGGAAAA---------
>Ca5.4 GenBank number: AR406141.1
ATGCGCTGTCTCCCAGTCTTCATCATTCTTCTGCTGCTGATTGCATCTGCACCTGGCGTTGATGCCCAAC
CGAAGACCAAATATGATGCGCCCCTGACATCTCTCCACGATAATGCAAAGGGTATACTACAAGAACATT
GGAACAAACGC---TGCTGCCCCAACAAG---CCTTGCTGTATAGGAAAA---------
>Mi5.1 GenBank number: JX293486
ATGCGCTGTCTCCCAGTCTTCATCATTCTTCTGTTGCTGATTCCATCTGCATCTAGCGTTGATGTCCAAC
CGTTGACCAAAGATGATGTGCCCCTGGCATCTTTCCTCGCTAATGCAAGACGAACCCTACAAAGTCTC
TGGATGACACGCAGGTGCTGTCCAAAAAAACCTTATTGCTGTCCAGGAAAA---------
>PnMRCL-B01122 GenBank number: AF214978.1
ATGCGCTGCCTCCCAGTCTTCGTCATTCTTCTGCTGCTGATTGCATCTGCACCAAGCGTTGATGCCCGA
CCGAAAACCAAAGATGATATACCCCTGGTATCTTTCCAAGATAATGCAAAGCGAGCCCTGCAAATACTT
TCGAACAAACGCTATTGCTGCTACTTCGACTATTCGTGCTGTAGA---------------
>PnMRCL-B01121 GenBank number: AF214976.1
ATGTGCTGCCTCCCAGTCTTTGTCATTCTTCTGCTGCTGATTGCATCTGCACCTAGCGTTGATGCCCTAC
CGAAGACCAAAGATGATATGTCCCTGGCATCTTTCCATGATAATGCAAAGCGAACCCTGCAAATACTTT
CAAACAAACGCTATTGCTGCGTCTACGACTATTCGTGCTGTGGA---------------

>TxMRCL-D022 GenBank number: AF214963.1
ATGCGCTGCCTCCCAGTCTTTGTCATTCTTCTGCTGCTGATTGCATCTACACCTAGCGTTGATGCCCGAG
CGAAGACCAGAGATGATATGTCCCTGGCATCTTTCCACGATGATGCAAAGCGAATCCTGCAAATACTTC
AGGACAGATCTGGTTGCTGCGTAATTGATAGCAACTGCTGTGGG---------------
>TxMRCL-D021 GenBank number: AF215096.1
ATGCGCTGCCTCCCAGTCTTTGTCATTCTTCTGCTGCTGATTGCATCTACACCTAGCGTTGATGCCCGAG
CGAAGACCAGAGATGATATGTCCCTGGCATCTTTCCACGATGATGCAAAGCGAATCCTGCAAATACTTC
AGGACAGATCTGGTTGCTGCGTAATTGATAGCAACTGCTGTGGG---------------
>PnMRCL-B01131 GenBank number: AF214954.1
ATGCGCTGCCTCCCAGTCTTCGTCATTCTTCTGCTGCTGATTGCATCTGCACCAAGCGTTGATGCCCGA
CCGAAAACCAAAGATGATATACCCCTGGTATCTTTCCAAGATCATGCAAAGCGAATCCTGCAAACATTT
GAGAGCAGATATGATTGCTGCAAAACA---TTCGAATGCTGTCATTGGGGA---------
>Tx5.15 GenBank number: JX293481
ATGCGCTGCCTCCCAGTCATCGTCATTCTTCTGCTGTTGATTGCATCTGCGCCTAGCGTTGACGCCCAA
CCGAAGACCAAAGATGATATACCCCAGGCATCTTTCCTAGATAATGCGAAGCGATACCTGCAAGTACTT
GAGAGCAAAAGAAATTGCTGCAGAAGG---CAAATTTGCTGTGGGAGAAAT---------
>TeAr193 GenBank number: DQ141141.1
ATGCGCTGTCTCCCAGTCTTCGTCATTCTTCTGCTGTTGATTGCATCTGCGCCTAGCGTTGACGCCCAA
CCGAAGACCAAAGATGATATACCCCAGGCATCTTTCCTAGATAATGCGAAGCGATACCTGCAAGTACTT
GAGAGCAAAAGAAATTGCTGCAGAAGG---CAAATTTGCTGTGGGAGAACAAAA------
>S5.1 GenBank number: JX293482
ATGCGCTGTCTCCCAGTCTTCGTCATTCTTCTGCTGCTGATTGCATCTGCACCTAGCGTTGATGCCCAAC
CGAAGACCAAAGATGATGTGCCCCTGCCACATTTGCACGATAATATACAGAATACTCTACAAACACTTC
GGAAGAAAGTC---TGCTGCCGCCCGATGCAGGATTGCTGTTCAGGGAAA---------
>Eb5.1 GenBank number: JX293519
ATGCGCTGTTTCCCAGTCTTCGTCGTTCTTCTGCTGCTGATTGCATCTGCACCTAGCGTTGATGCCCAAC
CGAAGACCAAAGATGATGTGCCCCTGGCACCTTTGCACGACAATATACAGAATACTCTACAAACACTT
CGGAAGAAAGTC---TGCTGCCGCCCGATGCAGGATTGCTGTTCAGGGAAA---------
>Gm5.2 GenBank number: AF167166.1
ATGCGCTGTCTCCCAGTCTTCGTCATTCTTCTGCTGCTGATTGCATCTGCACCTAGCGTTGATGCCCAAC
CGAAGACCAAAGATGATGTGCCCCTGGCACCTTTGCACGATAATATAAGGAGTACTCTACAAACACTT
CGGAAGAAAGTC---TGCTGCCGCCCAGTGCAGGATTGCTGTTCAGGGAAA---------
>Lp5.1 GenBank number: AY591769.1
ATGCGCTGTGTCCCAGTCTTCATCATTCTTCTGCTGCTGATTCCATCTGCACCCAGCGTTGATGCCCAAC
GGAAGACCAAAGATGATGTGCCCCTGGCATCTTTCCATGATAATGCAAAGCGAACCCTGAAAAGACTT
TGGAACAAACGCTCGTGCTGCCCACAAGAATTTTTATGCTGTCTATACCTGGTGAAA---
>Mr5.3 GenBank number: AY591767.1
ATGCGCTGTGTCCCAGTCTTCGTCATTCTTCTGCTGCTGATTGCATCTGTACCTAGCGTTGATGCCCAAT
TGAAGACCAAAGATGATATGCCCTTGGCATCTTCCCACGCTAACGTAAAGCGGACCCTGCAAATACTT
CGGAACAAACGC---TGCTGCATTACATTCGAATCGTGCTGTGAGTTTGACTTAAAA---
>Mr5.1a GenBank number: AY591764.1
ATGCGCTGTGTCCCAGTCTTCGTCATTCTTCTGCTGCTGATTGCATCTGCACCTAGCGTTGATGCCCGAT
TGAAGACCAAAGATGATATGCCCTTGCCATCTTCCCACGCTAACATAAAGCGAACCCTGCAAATACATC
GGAACAAACGC---TGCTGCCCAGGCTGGGAATTGTGCTGCGAGTGGGATGAGTGG---

>Mr5.4a GenBank number: AY591768.1

ATGCGCTGTGTCCCAGTCTTCGTCATTCTTCTGCTGCTGATTGCATCTGCACCTAGCGTTGATGTCCAAT
TGAAGACCAAAGATGATGTGCCCTTGGCATCTTTCCACGCTAACGTAAAGCGAACCCTGCAAATACTT
TTGAACAAACGC---TGCTGCCAAGTAATGCCACAGTGCTGTGAGTGGAAT---------

>Mr5.2 GenBank number: AY591766.1

ATGCGCTGTGTCCCAGTCTTCGTCATTCTTCTTCTGCTGATTGCATCTGCACCTACCGTTGATGCCCAAC
TGAAGACCAAAGATGATATGCCCTTGGCATCTTTTCACGCTAACGTAAAGCGAACCCTGCAAATACTTC
GGGACAAACGCTTCTGCTGCCGAACACAGGAAGTGTGCTGTGAAGCGATTAAAAATGGA

>lt5a GenBank number: DQ345351.1

ATGCGCTGTCTCCCAGTCTTCATCATTCTTCTGCTGCTGATTCCATCTGCACCCAGCGTTGATGCCCAAC
GGAAGACCAAAGATGATGTGCCCCTGGCATCTTTCCATGATAATGCAAAGCGAACCCTGAAAAGACTT
TGGAACAAACGCTCGTGCTGCCCACAAGAATTTTTATGCTGTCTATACCTGGTGAAA---

>Lt5e GenBank number: DQ345354.1

ATGCGCTGTCTCCAAGTCTTCATCATTTTTCTGCTGCTGATTCCATCTCCACCCAGCGTTGATGCCCAAC
GGAAGACCAAAGATGATGTGCCCCTGGCATCTTTCCATGATAATGCAAAGCGAACCCTGAAAAGACTT
TGGAACAAACGCTCGTGCTGCCCACGAGAATTTTTATGCTGTCTAAGA------------

>Lt5c GenBank number: DQ345352.1

ATGCGCTGTCTCCTAGTCTTCATCATTCTTCTACTGCTGATTCCATCTGCACCCAGCGTTGATGCACAAC
CGATGACCAAAGATGATGTTCCCCTGTCATCTCTCCATGATAATGCAAAGCGAGCCCTACAAATGTTTT
GGAACAAACGCGATTGCTGCCCAGCAAAAATGTTCTGCTGTCAATGG------------

>Lt5d GenBank number: DQ345353.1

ATGCGCTGTCTCCCAGTCTTCATCATTCTTCTGCTGCTGATTCCATCTGCACCCAGCGTTGATGCCCAAC
CGACGACCAAAGATGATGTGCCCCTGGCATCTCTACATGATAATGCAAAGCGAGCCCTACAAATGTTTT
GGAACAAACGCGATTGCTGCCCATCAAAACTTTTATGCTGTAATCCA------------

>Vn5.1 GenBank number: AF214964.1

ATGCGCTGTCTCCCAGTCTTCGTCATTCTTCTGCTGTTGATTGCATCTGCACCTGGCGTTGATGCCCAAC
CGAAGACCAAATATGATGTGCCCCTGGCATCTCGCCACGATTTTGCGAAGAAAACCCCAAAAAGACTT
TCGAAACCACGTGACTGCTGCCGCAGAAATTTCTTATGTTGT-----------------

>Vn5.2 GenBank number: AF214966.1

ATGCGCTGTCTCCCAGTCTTCGTCATTCTTCTGCTGTTGATTGCATCTGCACCTGGCGTTGATGCCCAAC
CGAAGACCAAATATGATGTGCCCCTGGCATCTCGCCACGATTTTGCGAAGAAAACCCCAAAAAGACTT
TCGAAACCACGTGACTGCTGCCTCAGACATTTCTTATGTTGTGTC---------------

>Vn5.3 GenBank number: AF214967.1

ATGCGCTGTCTCCCAGTCTTCGTCATTCTTCTGCTGTTGATTGCATCTGCACCTGGCGTTGATGTCCAAC
CGAAGACCAAATATTATGTGCCTCGGGCATCTCGCCGAGATTTTGCGAAGAAAACCCCAAAAAGACTT
TCGAAACTACGTGGCTGCTGCCCCCGAAGTTTCTTATGCTGTCGC---------------

>TxMRCL-011 GenBank number: AF214962.1

ATGCGCTGTTTCCCAGTCTTCATCATTCTTCTGCTGCTAATTGCATCTGCACCTTGCTTTGATGCCCGAA
CGAAGACCGATGATGATGTGCCCCTGTCACCTCTCCGCGATAATCTAAAGCGAACGATACGAACACGC
CTGAACATACGCGAGTGCTGCGAGGATGGA---TGGTGCTGTACTGGTCGT---------

>TxMRCL-012 GenBank number: AF214958.1

ATGCGCTGTTTCCCAGTCTTCATCATTCTTCTGCTGCTAATTGCATCTGCACCTTGCTTTGATGCCCGAA
CGAAGACCGATGATGATGTGCCCCTGTCATCTCTCCGCGATAATCTAAAACGAACGATACGAACACGC
CTGAACATACGCGAGTGCTGCGAGGATGGA---TGGTGCTGTACTGGTCGT---------

>S5.2 GenBank number: JX293483

ATGCGCTGTCTCCCAGTCTTCATCATTCTTCTGCTGCTAATTGCATCTGCACCTTGCTTTGATGCCCGAA
CGAAGACCGATGATGATGTGCCCCTGTCACCTCTCCGCGATAATCTAAAGCGAACGATACGAACACGC
CTGAACATACGCGAGTGCTGCGAGGATGGA---TGGTGCTGTACTGGTCGT---------

>PnMRCL-B01411 GenBank number: AF214983.1

ATGCGCTGTTTCCCAGTCTTCATCATTCTTCTGCTGCTAATGGCATCTGCACCTAGCTTTGATGCCCGAC
CGAAGACCGAAGATGATGTGCCCCTGTCATCTTTCCGCGATAATCTAAAACGAACCCTACGAACACTT
CTGGACCCACGCAGGTGCTGCTATGAAACTCCAGGGTGCTGTGTTATTGGA---------

>PnMRCL-0111 GenBank number: AF214975.1

ATGCGCTGTCTCCCAGTCTTCATCGTTCTTCTGCTGCTAATTGTATCTGCACCTGGCTTTGATGCCCGAC
CGAAGACCGAAGATGATGTGCCCCTGTCATCTTTCCACGATGATCTACAGCGAACCGTACGAACACTT
CTGGACATACGCATGTGCTGCCTTGGTACTTCAGGGTGCTGTCCTTGGGGA---------

>Tr5.2 GenBank number: JX293491

ATGCGCTGCCTCCCAGTCTTCATCATTCTTCTGCTGCTGATTCCATCTGCACCTAGCACTGATGTCCAAC
CGAAGACCAAAGATGATGTGCCCCTGGCATCTTTCCGTGATAGTGTAAAGCGAATCCTACAAAGACAA
---------------TGCTGCCCAACGATTTCTGAGTGCTGTCGTGTAGGA---------

>Tr5.1 GenBank number: JX293490

ATGCGCTGTCTCCCAGTCTTCATCATTCTTCTGCTGCTGATTCTACCTGCACCTAGCGCTGATGTCCAAC
CGAAGACCAAAGATCATGTGCACCTGGCATCTTTCCTTGATAGTGCAAAGCGAACCGTAAGAGGACAT
---------------TGCTGCCCATATTACCCTCAGTGCTGTCCTAGTGGA---------

>Vx5.1 GenBank number: JX293496

ATGCGCTGTCTCCCAGTCTTCATCATTCTTCTGCTGCTGATTCTACCTGCACCTAGCGCTGATGTCCAAC
CGAAGACCAAAGATCATGTGCACCTGGCATCTTTCCTTGATAGTGCAAAGCGAACCGTAAGAGGACAT
---------------TGCTGCCCATATTACCCTCAGTGCTGTCCTAGTGGA---------
